# A Sketch of the Life, and Some Account of the Works, of the Late Dr. James Johnson

**Published:** 1846-01

**Authors:** 


### MEDICO-CHIRURGICAL

### REVIEW.

JANUARY, 1846.

### A Sketch of the Life, and some Account of the Works, of the late Dr. James Johnson.

Multis ille bonis flebilis occidit;
Nulli flebilior quàm mihi.

On the 10th of last October, died Dr. James Johnson. This Review owed its existence to him. It originated in his bold conception—it was raised to a high pitch of circulation and celebrity, by his talents and industry—it was written with his own hand for nearly twenty years, and carried on under his watchful superintendence for more—it was regarded by him with a sort of paternal affection—and we should be wanting in all that gives dignity to gratitude, and gracefulness to affection, if we did not, to the best of our poor abilities, set forth his claims to the respect of the Profession, and to be ranked with its illustrious dead.

Nor are those claims slight. If natural ability, varied information, industry that never quailed, a ready pen, a caustic and yet a kindly humour, professional knowledge acquired under every difficulty and dispensed with unbounded generosity, a probity that could not swerve, and a benevolence that knew no limit to its objects, constitute the features of a character to be admired as well as loved, then the subject of this Memoir will be both.

James Johnson, or rather Johnstone, for that was really his name,* was born in February 1777, on the banks of Lough Neagh, in the parish of Ballinderry, County of Derry, Ireland. Like Cobden, he might boast that he was "a farmer's son," his father cultivating a small farm, which had

* An accident or a whim led originally to a change, which prudential considerations perpetuated. Perhaps, such may no longer influence his family, who may choose to resume their real surname.

been for some time in the possession of the family. James Johnson was the youngest of the children, all of whom he lived to survive; none of them distinguishing themselves sufficiently to require any further notice.

At the age of six years, he was put to a grammar-school, kept by a Catholic, the village pedagogue, and brother of the parish priest. Whatever his attainments, they were probably of no very elevated order, for the pupil in after-life spoke somewhat lightly of them, and implied, at all events, if he did not state, that he learnt little at school, which he cared subsequently to remember. At the time, however, he thought differently—he always sought to be, and generally was, at the head of his class—he was miserable, as he himself tells us, when not so—and he would sit up till midnight conning the lessons of next day. The boy is the type of the man, and the seeds of those qualities, good or bad, which shall ripen in our maturity, are sown and germinate in our most tender years. Ambition, "the last sin of noble minds," will reign in the breast of the village lad, and, just as it is well or ill directed, will lead its slave to reputation or to ruin. It conducted young Johnson to the former, thanks to a mind naturally strong, and to a moral sense implanted early, cultivated always, and confirmed rather than shaken by the struggles of life.

Before he was fifteen he had quitted school, and the instruction that he got there was all that he ever obtained from others. Great as were his acquirements in later years, and various as his reading, they were gained when the age of pupillage and almost that of youth was over, and were solely the fruit of his own application and his thirst for knowledge. He carried with him from the school-room of Ballinderry, a moderate acquaintance with the construction of his own language, a still more moderate idea of Latin, a notion that Greek was something altogether beyond his reach, and a total ignorance of every modern foreign tongue.

At the age of fifteen, he was apprenticed to Mr. Young, a surgeon-apothecary in Port-Glenone, in the County of Antrim. He remained there only two years, when he was transferred to Mr. Bankhead, of Belfast, where he continued for two years more, and then came to London, without either money or friends. He became assistant to an apothecary in the Metropolis, and, by hard study and irregular attendance on lectures in anatomy and surgery, he passed a creditable examination at Surgeon's Hall, in 1798, and was appointed surgeon's mate in the navy, in the month of May of that year. In the Mercury frigate, he sailed to Newfoundland and Nova Scotia, always reading indefatigably, and seizing every opportunity, when the ship was in harbour, to visit the naval hospitals, to watch the cases they contained, and store up professional knowledge. Now, and ever after, he made notes of any thing important that he saw, and is another instance (if instances were wanting) of the value of recording facts and impressions, at the time of their occurrence.

Captain Rogers, the Commander of the Mercury, had a great antipathy to the Irish, but the zeal and good conduct of the young surgeon's mate subdued the ungenerous prejudice, and Captain Rogers winked at his absence from the ship, for some months, in the Winter of 1799, when he studied night and day in London. The fruits of his diligence were soon apparent, for, in January 1800, he passed, for the second time, a triumphant examination at Surgeon's Hall.

He had obtained some honour, and, what was more substantial, he had gained a patron in Captain Rogers. This gentleman, of whom he always spoke in terms of affectionate remembrance, got him made full surgeon, and appointed to the Cynthia, sloop-of-war, on the 27th of February. He was now in the twenty-second year of his age, and, in the Cynthia, he accompanied the famous expedition to Egypt, was at the siege of Belleisle, and at all the descents which the troops made on the coasts of France, Spain, &c. till they reached their destination. The fatigues and anxious nature of his duties proved too much for him; he was attacked with illness, and was sent back to Gibraltar, where he did duty for some time, under Mr. Vaughan, the Surgeon of the Naval Hospital.

From Gibraltar he returned to London, in the Winter of 1800. He had recruited from his sickness—saved a little from his pay—had a few months before him—and resolved to expend all that he possessed, both in guineas and in time, in working out that professional education, which had so far been acquired, and must now be completed, by hurried and broken snatches. The Sybaritic student of the present day, propped up on all sides by prosectors and by lecturers—provided with means, and glutted with help—who flies, in despair, at the difficulties that overwhelm him, to the friendly arms of the *Grinder*, may blush, perhaps, to read how Mr. Johnson struggled to obtain an education, unsupported by money, unassisted by friends, unaided by those manuals, plates, and woodcuts, which, professing to facilitate, appear to supersede exertion, and form at once the refuge and the ruin of complaining indolence. But "heaven helps those who help themselves," and so thought, or seemed to think, the subject of this Memoir.

He went to the Anatomical Theatre in Great Windmill Street, and placed himself under Mr. Wilson and Mr. Thomas. During the Winter he distinguished himself as a dissector, and generally prepared the subjects for his teachers' lectures—an opportunity, which once was sought with eagerness and regarded as a prize, but seems now to be mostly considered a *bore*. It was in this Winter that the present Master of the Rolls, Mr. Bickersteth, and Mr. Johnson, formed, with others, a society of six, who gave demonstrations daily, in their turn, to a large class of students in the Theatre.

In this way the Winter went, and with it went also Mr. Johnson's means. He had expended his last farthing, and midwifery lectures were yet to be attended. A fee was to him an impossible thing. Dr. John Clarke was the midwifery teacher, and to him Mr. Johnson frankly stated his circumstances. That distinguished Accoucheur immediately presented him with a ticket of admission, and asked him to his table. A liberal and generous spirit is hereditary in the Clarke family. The anecdote was often told when the needy pupil had become the affluent physician.

In June, 1801, Mr. Johnson, whatever his thirst for knowledge, discovered that the lamp must be fed with oil, and was warned by his circumstances to seek employment. He applied to the Navy Medical Board for a ship, and his best credentials were contained in a note by Mr. Wilson :— "The *bearer* of this, Mr. James Johnson, has actually *lived* in the dissecting-room of Great Widmill Street during the last six months. Examine him, and see whether he has studied in vain." Dr. Harness was at the head of the Board, and, to his credit, he instantly appointed the young anatomist, devoid as he was of friends and interest, to the "Driver" sloop-of-war. In this ship he served in the North Sea, visiting the Orkney and Shetland Islands, and going with a convoy to the vicinity of Greenland, and Hudson's Bay.

At the peace of 1802, he was again out of employ. Many young men would have done nothing, or have done mischief. They would have "seen life," that is, mis-spent or sacrificed it. Mr. Johnson's mode of passing leisure time was a different one. His economical habits had again enabled him to save a few pounds, and he put it out at interest in—*study*. So soon as his money was gone, something must be done. To get on the peace establishment was very difficult, for it required great interest, and he had none. He applied to the Sick and Hurt Board, and was fortunate enough to obtain a conversation with the late Sir Gilbert Blane. Like Dr. Harness, Blane was pleased with the ingenuousness, enthusiasm, and industry of the applicant, and he immediately appointed him to the "Caroline."

He now gave indications of his taste and talent for writing, and at the age of 27, amidst the bustle and confusion of a mess-room, and surrounded by his brother-officers, men whose gallantry was more unquestionable than their literature, he composed his earliest work. It could hardly be on any professional subject, for of professional experience he had had little—and it would seem difficult to extract from a sea-voyage the materials for an octavo volume. But,

Nil mortalibus arduum est, and it would be a dull journey indeed, of which the fancy and industry of Johnson could not make a lively sketch.

The work itself, which now lies before us, bears the following, not altogether uncharacteristic, title :—

"THE ORIENTAL VOYAGER; OR DESCRIPTIVE SKETCHES AND CURSORY REMARKS, ON A VOYAGE TO INDIA AND CHINA, in his Majesty's Ship Caroline, performed in the years 1803-4-5-6, interspersed with Extracts from the best Modern Voyages and Travels. The whole intended to exhibit a topographical and picturesque Sketch of all the principal Places which are annually or occasionally visited by our East India and China Fleets. The Routes to and from India, illustrated by the tracks of His Majesty's Ships Caroline and Medusa, correctly set off on a Chart, extending from the British Isles to Canton. By J. JOHNSON, Esq. Surgeon in the Royal Navy.

"Et mores hominum multorum vidit et urbes." HOR.

"Wandering from clime to clime, observant stray'd,
"Their manners noted, and their coasts survey'd." ODYSSEY.

London: 1807."

If there is anything which distinguishes Dr. Johnson as an author, it is his knack at giving a name to his books. Simplicity is not the great feature of his title-pages, nor yet are they merely elaborate advertisements, but there is a quaint affectation about them, indicative at once of a turn for the poetical, and of the absence of severe taste. The Oriental Voyager—the Influence of Tropical Climates on European Constitutions—Morbid Sensibility of the Stomach and Bowels—the Stream of Human Life—the Pilgrim of the Spas—are all specimens of the not infelicitous, though, perhaps, not altogether unobjectionable, names, which he gave to his literary offspring.

The Preface gives a tolerably accurate account of the nature of the volume. His object, he informs us, is to furnish the young voyager with an agreeable and useful companion on his first visits to the Oriental World.

He apologises for the style, hinting that the maritime adventurer is precluded not only from a liberal communication with books, but confined for the most part to a limited and peculiar class of society—he fears that the critic (to whom he pays a compliment more just than common, a compliment, perhaps, based on a prophetic consciousness that himself would rank high in the class hereafter), may detect some inaccuracies and much roughness—he deprecates censure on the natural ground that he had been compelled to write in a desultory manner, often on a rude and boisterous element, and continually interrupted by professional pursuits—and he winds up his first preface, as, in after life, he was apt to do many a page, with a poetical quotation :—

"Far from the Muses' academic grove,
'Twas his, the vast and trackless deep to rove.
Alternate change of climate had he known,
And felt the fierce extremes of either zone,
Where polar skies congeal th' eternal snow,
Or equinoctial suns for ever glow:
From regions where Peruvian billows roar
To the bleak coast of savage Labrador."

Falconer's Shipwreck might fairly be imagined to form part of the library of a sailor, himself not indisposed to worship at the altar of the Muses. In his subsequent writings will be found many passages taken from it, as well as from Pope's translations of the Iliad, and the Odyssey. It cannot be said of Johnson, as Pope said of himself, that he

Lisped in numbers, for the numbers came; but if he had not the inspiration, he imitated the manner and he admired the metre of the master.

Some account of his first work, unknown as it must be to the majority of those who became acquainted with his later ones, may not be altogether out of place. An author in his maturity is a different sort of person from one at his first start in life, and we feel as curious to see him in both aspects, as to look at the portrait of some great man at different ages.

Mr. Johnson tells us that he sailed from Cork in May, 1803, in His Majesty's ship Caroline, of 36 guns. She bore, in sealed orders, the declaration of war against France, and had orders to detain all vessels belonging to the Batavian Republic. The first place at which they touched was Madeira, and, as that Island still has claims on our attention, we are disposed to introduce Mr. Johnson's earliest impressions of it. As a London physician, we know that he thought highly of it, and sent many consumptive patients to it.

" At day-light this morning, we found ourselves close in with the north-east point of Madeira; and as the sun arose, the whole prospect of Funchal, and the surrounding villas, churches, &c. burst upon our view. This bay has a truly romantic and beautiful appearance. The town (the houses of which are all white and look remarkably well) lies at the bottom of the bay; and the ground forming the extremities of the latter, rises at first with a gradual, and afterwards with a very steep ascent, in the form of an amphitheatre. From the sea up the steep part, the whole is covered with vineyards, villas, orangeries, churches, and convents, rising in gradation, and forming a most picturesque landscape; while the steep cliffs, raising their fantastic and wood-clad summits above the clouds, majestically crown the whole."

They found, on entering the town, that it was like most other Portuguese cities, handsome enough *outside*, but disgusting *within*.

" The streets were narrow and dirty; the houses high and inconvenient; with the inhabitants corresponding, ragged though tawdry, and dirty though proud. Englishmen in general, when they get into a Catholic country, immediately visit the convents, monasteries, and churches; not, I believe, through any particular veneration for religion, but sometimes to satisfy an idle curiosity; or perhaps (which is worse) to have a sneer at their superstition. However that may be, we left very few places of the above description unexplored. They seem very glad at the convents to see an Englishman; when they immediately exhibit their artificial flowers, and other curiosities, which he buys at an exhorbitant price: for, however the English may be excelled in gallantry by their more polite neighbours, yet, when pecuniary affairs are on the carpet, I'll answer for it they will have the preference even among the fair sex."

After giving an account of the appearance and manners of the natives, with other matters which, though amusing, are irrelevant to our present purpose, Mr. Johnson makes a brief remark on the climate and salubrity of the island. After observing that a great number of invalids with pulmonary complaints resort to it, he states:—

" It is not, however, exempted from fevers and other continental diseases; for I was told by an English physician, a resident on the island, that during the months of September and October, 1802, it had been visited by the same epidemic catarrhal fever which made such ravages in the months of December, January, and February following, in England and on the continent."

The epidemic influenza did not spare Madeira, and we may safely say, that wherever there is a humid atmosphere, and vicisitudes of temperature occur, a febrile catarrh will never be entirely unknown.

We shall not pursue the voyage very circumstantially. At 1 p. m. of the

6th July, 1803, the ship crossed the Line, and we have an account of the ceremonies on that eventful occasion, drawn, of course, from the life.

"A particular and very careful list of all those who cannot give satisfactory proofs of having crossed the *line* before, is made out; they are then confined in the 'tween decks, and brought up one by one into the *waist*, where the apparatus and performers are ready to receive them; none, however, being permitted to return below after the ceremony, lest they should give hints to their companions, that might prove detrimental to the succeeding operations. The dresses of Neptune and his train, on this occasion, are truly grotesque; long half-wet *swabs*, bespattered with flower or oatmeal, compose their flowing locks, while their faces are bedaubed with red ochre, and other colours, that make them appear like deities of a still lower region than the sea! A large grog-tub, filled with *salt water* is now placed under one of the gangways, with a stick crossing it, in such a manner as to be easily made to slide into the water occasionally; on this, the man to be shaved is placed, and the barber (who has previously mixed up a potful of tar, soot, blacking, dirty grease from the galley, and some other ingredients that shall be nameless) begins to ask him some question or other, which the poor *novice* no sooner opens his mouth to answer, than he has the brush thrust in, and in fact finds himself instantly lathered from ear to ear with this odious composition! A piece of iron hoop notched with a file, and as rough as a saw, now serves the place of a razor; with which being shaved, or rather, most woefully scraped, the signal is given, the seat gives way, and down he tumbles into the tub of water; when perhaps thirty or forty buckets are kept bailing on him from the boats and booms; till at length, after struggling and plunging till he is half drowned, the poor wretch is liberated from the watery ordeal."

They now ran along the Coast of Brasil—on the 2nd of August, they captured a couple of Dutchmen, one laden with a rich cargo—a sketch of Cape Town, of some vigour and interest is presented to us,—and we have a rationale of the *Table Cloth*, that fleecy cloud which clothes the summit of Table Mountain, and is watched with much curiosity by both native and stranger.

Apoplexy and diseased liver, with its consequence, dropsy, are common. Instances of longevity are rare, few exceeding the age of sixty. We are induced to make so long an extract from the circumstance that the Cape has lately attracted some attention from its reputed and reported salubrity. The records both of the army and navy lead to this conclusion, and the mortality of the settlement would not seem, so far as our troops are concerned, to exceed that of Great Britain itself, if it is not actually below it.

A trait which early and always distinguished the writings of Johnson is their liveliness. If an observation is not profound, it is pretty sure to be happy, and, however quickly made, it is rarely devoid of sagacity and penetration. This feature contributed, as much as any, to render his works so popular. In the volume before us we are continually struck by the contrast between the solemn dulness of a quotation from other travels, and the vivacity of the author's own remarks. Take a sample:

"In contemplating the manners and opinions of different nations, we are often apt to attribute to the caprice of the human mind, effects which proceed from natural causes alone, over which man can scarcely be allowed to possess any influence at all.

"The cleanliness and industry of the Dutch form a striking contrast with the dirt and indolence of the Portuguese, but are not more opposite than the climates of Holland and Portugal. The religious sentiments of these two nations are not less different than their external manners, and may, perhaps, be ultimately deduced from the same cause.

"At Rio Janeiro, the lofty spires of innumerable churches arise in every point of view; the streets are crowded with priests of every denomination and habit: the air continually reverberates the solemn sounds of the cloister bell, while the harmonious notes of the vesperal hymn, chaunted in slow cadence, break the silence of the evening, and forces reverence from the breast of levity itself.

"At the Cape of Good Hope, two churches and two clergymen are enough for the inhabitants; and at Simmon's Town, there is no trace of the peculiar appropriation of the Sabbath to religious duties: all here are employed in making money. Money is the supreme divinity of a Dutchman, for which he would renounce his religion, sell his wife, or betray his friend!"

From the Cape they steered to Ceylon, and on the 4th of September they saw the fires on its mountains. They had now run upwards of thirteen thousand miles, and made a voyage of one hundred and four days, with only the preparation for a cruise in the Channel. Yet they had not lost a man by sickness, and there had not appeared a single symptom of scurvy. Mr. Johnson attributes this to the discipline and cleanliness of the ship's company. "As to anti-scorbutics," he says, "there were only two or three cases of lime-juice on board the ship; which could not be of much consequence among 264 men. Much, indeed, I think, depends on keeping the men's minds employed, during long voyages, in little amusements and recreations, which are not at all incompatible with good discipline: every fine afternoon, therefore, the dance commenced under the half deck or gangways, which was kept up till eight o'clock, diffusing a general exhilaration of spirits through the whole of the crew."

After casting anchor for a few hours in Trincomallee harbour, the Caroline set sail for Madras, and on the morning of Sept. 8, she was in the Roads. On the 10th, they set sail for Bengal, and on the 24th, they anchored in the Hoogly. We are not aware if provisions continue as reasonable as they were in 1803. Then, three or four pine-apples cost only an ana, or 2d English, and other things in proportion. Fowls and ducks two rupees, or 5s. per dozen; geese three rupees, or 7s. 6d. ditto; and all other species of stock equally cheap.

The river is, or was, greatly infested with alligators, of which Mr. Johnson gives a lively account.

" A creek about a mile to the northward of the village, has been the haunt of one, who for many years has rendered himself formidable to the neighbourhood, by his depredations and enormous size, being, it is said, 28 or 30 feet in length. Two of us having landed late in the evening at Kedgeree, found it very difficult to prevail on some of the villagers to accompany us across this creek, to Mr. Jackson's, the English resident, who lives about two miles from thence. On our way along the banks of the river, we at one time, near this creek, heard a rustling noise among the jungle; at which our guides seemed so much affrighted, that they were on the point of taking to their heels, and leaving us to find our way as we could. We did not know the cause of this panic until we got to Mr. Jackson's; when we were informed, that only two nights before this, a man had been destroyed by an alligator at the very spot where we heard the rustling noise. Some time after this, I purchased a young one, about four feet in length, from a fisherman who had caught it in his net. Its figure exceedingly resembles the guana; and it likewise bears a considerable similitude to the lizard: it could run but slowly along the decks, with its lower jaw close to them; and on presenting a stick, it would snap at, and lay hold of it very readily. The extent to which it would open its mouth on these occasions, could not possibly be effected by the falling of the lower jaw alone, which, as I said before, it kept nearly in contact with the decks: the two jaws therefore, in this operation, seemed to recede from each other like the blades of a pair of scissors when opening. As I conceived that this appearance might possibly give rise to the old opinion, that the upper-jaw of the crocodile was moveable, I examined particularly the head of this one after its death. In the first place, there was no joint or motion between the upper-jaw and the head, as the Jesuits at Siam, who dissected this animal, have justly remarked: but they have not (if I recollect right) taken notice of any peculiarity in the lower jaw's articulation with the bones of the head, which is different from that of any other animal with which I am acquainted. Here, instead of the *head of the under jaw-bone being received* into a cavity in the bones of the skull, (as I believe is generally the case,) it was, on the contrary, *hollowed out*, to receive an articulating process from the skull; as if the former was meant to be the fixed point, and the latter the moveable one. The fact is, that when the animal is opening his mouth to any great extent, while the lower jaw falls, the strong muscles on the back of the neck *draw backwards the head, and raise the upper-jaw at the same time:* this, in all probability, first suggesting the idea of the mobility of the crocodile's upper jaw. Here, as usual, nature has artfully adapted the structure to the peculiar functions of the animal. The alligator, whose legs are very short, and whose jaws are uncommonly long, (perhaps one-eighth of his whole length,) would not, when on shore, be able to open his mouth to half its natural extent, if the motion depended on the under jaw alone: for, owing to the lowness of the animal's body and head, this jaw would come in contact with the ground before the mouth was sufficiently extended; and therefore nature has given it the power of raising the upper jaw occasionally with great ease.

"It is an erroneous opinion that this animal's back-bone is not sufficiently flexible to allow of his turning short, when in pursuit of his prey; and that therefore a man, by taking a winding course, when pursued, might easily elude him. I would not advise any one to trust to this manœuvre; though I believe the alligator seldom attempts to seize any creature otherwise than by surprise: for this purpose he frequently lies among the mud on the shores of this river, or in the creeks that open into it, and when any animal is passing near him, he is almost sure of securing him, on account of the great length of his destructive jaws. He frequently too throws himself across the boats that are hauled up into these creeks, and tears the poor defenceless fisherman to pieces in an instant, or dives to the bottom of the river with him, where he devours him at his leisure. Dogs, especially of the Paria kind, and jackals, that come down to the edge of the river to drink, very often fall sacrifices to the insidious alligator; who will lie close to the banks, and at those times very much resembles the trunk of a tree, or pieces of floating wreck. It is said that, when in pursuit, (which however is seldom the case,) he generally endeavours to get abreast of the object, and then, by making a sweep with his extensive jaws, he seldom fails to secure his victim. The teeth of this animal are terrible to behold; long, sharp, and inter-locking with each other, evincing his being solely carnivorous: besides which, there are two in the front of the lower jaw, longer than the rest, and which pierce through the upper jaw, coming out at two apertures near the nostrils; so that, having once laid hold of his prey, there is little chance of its being able to extricate itself afterwards from such engines of destruction."

Mr. Johnson compares the rice-diet of the Hindoo with that of the European in the East, and hints, that if the latter were to relax somewhat from beef-steaks in the morning, a sumptuous dinner at seven, and a bottle of wine afterwards, he might possibly avoid a few of those Oriental disorders which are fastened too readily upon the climate.

In October the frigate sailed for Rangoon, where the crew first made the acquaintance of the *prickly-heat*. The sensations arising from this are indescribably tormenting: it is next to an impossibility to avoid rubbing or scratching the part where it first is felt; and this action setting the body in a glow, the merciless prickly heat attacks every part, goading one almost to madness with its infernal stings. Nor is there any cure for this singular affection of the skin but patience, and keeping one's self as quiet and unruffled as possible: cold bathing, indeed, gives a temporary relief, but this interval is generally succeeded by a more virulent attack than before. It is said, that hair-powder dusted over the skin gives the most permanent ease.

In November they returned to Bengal, anchored again in the Hoogly, and found a great mortality prevailing among the Indiamen lying there. A sketch of the fever is backed by exhortations to temperance and continency, and an apt allusion from the Odyssey to those Syrens whom the navigator encounters in every port, and who charm but to betray on every shore.

> "Their touch is death, and makes destruction please.
> Unblest the Youth whom Folly leads to stay
> Nigh the curst shore, or listen to their lay."

Among the lions of Calcutta was, and probably is, that fatal spot, in the old fort called the *Black Hole*—a place about eighteen feet square, where, in 1756, the brutal Soubah of Bengal confined Mr. Holwell and 145 others, from eight o'clock in the evening till six the next morning. Most persons have heard of the Black Hole of Calcutta; many have a general notion of the horrible effects of confining many persons in it; but such a picture as this, physiological as well as striking, of the effects of extreme heat, and of an atmosphere deprived of oxygen upon the human body, is, we conceive, new to the majority of the present generation.

"While standing here, I could not help retracing, in imagination, the heart-rending scenes of that bloody tragedy, which fancy painted in glowing colours, while it execrated the monster who caused it to be perpetrated. The leading particulars of this horrid event are the following:—

"It was about eight o'clock at night when these unhappy persons were crammed together in a situation where no air could reach them, being open only to the West, by two windows, strongly barred with iron, and from which they could receive scarcely any circulation of fresh air. They had been but a few minutes confined, before a profuse sweat broke out on every individual, attended with an *insatiable thirst*, which became the more violent as the body was drained of its moisture. It was in vain that they stript off their clothes, or fanned themselves with their hats. A difficulty in breathing was next observed, and every one panted for breath. Mr. Holwell, who was placed at one of the windows, accosted the sergeant of the guard, and endeavoured to excite his compassion; he drew a pathetic picture of their sufferings, and promised to gratify him in the morning with a thousand rupees, provided he could find means to remove some of his people into another place of confinement.

"The Indian, allured by the promise of so mighty a reward, assured him that he would use his utmost endeavours, and retired for that purpose. What must have been the impatience at this time of these unhappy objects! In a few minutes the jemmidar returned; but the *tyrant*, by whose order alone such a step could be taken, was asleep, and no person durst disturb his repose! The despair of the prisoners now became outrageous; they endeavoured to force open the door, that they might rush on the swords of the monsters by whom they were surrounded, and who derided their sufferings; but all their efforts proved ineffectual. They then used execrations and abuse to provoke the guard to fire upon them. The jemmidar was at length moved to compassion; he ordered his men to bring some skins containing water, which, by enraging the appetite, only served to increase the general agitation; there was no way of conveying it through the two windows but by hats, and this mode of conveyance proved ineffectual, from the eagerness and transports of the wretched prisoners, who struggled for it in fits of delirium! The cry of water! water! issued from every mouth. The consequence of this eagerness was, that very little fell to the lot of even those that were nearest the windows; and even those who were esteemed the most fortunate, instead of finding their thirst assuaged, grew more impatient. The confusion soon became general and horrid; all was clamour and contest; those who were at a distance endeavoured to force their passage to the window, and the weak were pressed to the ground, never to rise again! Mr. Holwell observing now his dearest friends in the agonies of death, or dead, and inhumanly trampled on by the living, and finding himself wedged up so close as to be deprived of all motion, he begged, as the last mark of their regard, that they would, for one moment, remove the pressure, and allow him to retire from the window and die in quiet. Even in such dreadful circumstances, which might be supposed to level all distinctions, the poor delirious wretches manifested a respect for his rank and character; they forthwith gave way, and he forced his passage into the centre of the place, which was less crowded, because by this time, about one-third of the number had perished, while the rest still pressed to both the windows. He retired to a platform at the farther end of the room, and lying down upon some of his dead friends, recommended his soul to Heaven. Here his thirst grew insupportable; his difficulty in breathing increased; and he was seized with a strong palpitation of the heart. These violent symptoms, which he could not bear, urged him to make another effort; he forced his way back to the window, and cried aloud *Water! for God's sake!* He had been supposed already dead by his wretched companions; but finding him still alive, they exhibited another extraordinary proof of regard to his person. *Give him water!* they cried; nor would one of them attempt to touch it until he had drank! He now breathed more freely, and the palpitation of his heart ceased; but finding himself still more thirsty after drinking, he abstained from water, and moistened his mouth, from time to time, by sucking the perspiration from his shirt sleeves, which tasted soft, pleasant, and refreshing. The miserable prisoners now began to perceive that it was *air*, and not *water*, they wanted: they dropped fast on all sides; and a pungent steam arose from the bodies of the living as well as those of the dead, volatile as hartshorn.

" Mr. Holwell, being weary of life, retired once more to the platform, and stretched himself by the Rev. Mr. Bellamy, who, together with his son, a young Lieutenant, lay dead, locked in each other's arms! In this situation he was soon deprived of sense, and seemed to all appearance dead, when he was removed, by one of his surviving friends, to one of the windows, where the fresh air brought him back to life.

" The Soubah being at last informed that the greater part of the prisoners were suffocated, inquired if the chief was alive: and, being answered in the affirmative, sent an order for their immediate release, when *no more than twenty-three survived, out of one hundred and forty-six,* who entered this prison!!! Calcutta was re-taken the next year, and the inhuman Soubah was soon afterwards deposed, and *murdered* by his successor."

The ship now left Calcutta, and the crew suffered from dysentery and fever. Mr. Johnson himself was attacked by the former, and forty-two years afterwards, when sinking under the same complaint at Brighton, he referred to his illness in the Ganges, observing, that he little thought he should be carried off in England by what he had recovered from in India. Happily, the future is shut from our sight, or indifference and despair would often supersede those laborious efforts which procure for ourselves fortune and fame, advance civilization, and benefit our species. The sickness in the ship afforded Mr. Johnson an opportunity of observing the good effects of mercury in such disorders in hot climates, and this observation was the nucleus of his work upon that subject. Perhaps he rather overrated those effects, and contracted a predilection for the active exhibition of that dangerous, though valuable, drug, which was reduced to a juster estimate in later life.

From Saugur roads, the Caroline conveyed the fleet of homeward-bound Indiamen till abreast of the Andaman Islands, when she left them to pursue their voyage, chased a privateer, and took her. So shabby, however, was her conduct, that, in the most unhandsome manner, she poured a gratuitous broadside into the Caroline, in the act of hauling down her colours. Of course it was not returned.

Their destination was once more Bengal, and the return to it along the Malay coast, and the shores of the Andaman Islands, affords an opportunity for much varied and charming description. Another privateer was taken, and the Ganges reached, the ship lying in the river till the 8th of March, when a second fleet of Indiamen was conveyed to the Andamans, and the Caroline hauled up for Madras. The voyage, owing to baffling winds, occupied thirty-five days. From Madras to Masulipatam, and from Masulipatam to Madras, were trips that filled up the time till June, the only variety being scorching winds, suffocating clouds of dust, tempests, and thunder-storms, and an accident by which three men were drowned, and fourteen dreadfully mangled.

Early in August, 1804, orders were received to victual for six months, and take charge of the China convoy, then collecting at Madras. Three other men-of-war were added, as an attack from the French was apprehended. On the 13th, they set sail from Madras-roads—early in September they entered the Straits of Malacca—were again visited, and one of the fleet struck by, lightning—saw several water-spouts—and, on the 13th, anchored in Malacca Roads. The Malays, it is well known, are very dangerous, when under the influence of opium, they run *a-muck*, with their poniards, called kreeses, or kresses, stabbing every-one they meet.

" It is said these weapons are poisoned with the celebrated juice of the *Upas*-tree, but I believe very few of them have this property. I was once bargaining with a Malay for one of those kresses, which he said was deadly poisoned, and in drawing it out of the scabbard, cut myself between the fore-finger and thumb, at which I was not a little alarmed; an old man, however, who was standing by, opening a leaf of betel, took out a piece of *chunam* and applied it to the part: whether this had any effect or not I cannot tell, but I felt no more of the cut.

" It is probable that the greater number of their *kresses* are poisoned, merely by heating them red hot, and then plunging them into *lime juice;* the rust thus produced on the surface, and in the grooves of these weapons, leaves a most dangerous wound; not, however, so dreadfully fatal as the gum of the celebrated Upas-tree is said to be."

On the 16th of September the fleet started from Malacca, and in two days arrived at the straits of Sincapore. On the 22nd they quitted that, and passed a guano rock, Pedra Branca, where the Chinese seas commence. On the 2nd of October they were overtaken by a typhoon. A " great wind" (*Ta-fung*), indeed, it seems to have been, but it carried them to China notwithstanding, and on the 7th of October, after many hair-breadth 'scapes, we find them in the mouth of the Tigris river. While they lay here, in Lintin Bay, with a pleasant country, a delightful sky, and an ample supply of fish, flesh, fruit, and vegetable, the sick became more numerous every day. On this point, Mr. Johnson remarks:—

" The principal complaints among our seamen in China, were intermittent fevers, fluxes, and some liver complaints. We had often from sixty to eighty men at a time, unable to do duty at this island, though no particular cause appeared why we should be so unhealthy, unless it was occasioned by the sudden transition from an Indian climate to this one. There was only one small tank on this side of the island, which was otherwise hilly, and the soil dry and gravelly; the air was cool and agreeable, and very little rain fell during our stay here: we, nevertheless, became very sickly, as did the crews of the Indiamen at Wampoa; which last circumstance, indeed, is less to be wondered at, as Wampoa is surrounded by extensive marshy grounds and paddy-fields, which might tend to bring on intermittents and fluxes. But why we should be sickly, is not easily accounted for, as the Dedaigneuse frigate, which lay at Macao, thirty miles from us, continued perfectly healthy during the whole of our stay in China. Here, therefore, as in the Ganges, the higher up the river a ship proceeds, the more likely is she to become sickly.

"It may here be proper to remark that, in the Grampus and Caroline, when the bark was all expended on the numerous agues, and different kinds of intermittents that occurred, the Surgeons had recourse to calomel, which cured the diseases; but those who were cured in this manner, were almost invariably attacked with the same complaints again, when the influence of the mercury was completely gone off. This seldom happened with those who were cured by bark."

The European public has lately become acquainted in some measure with the Celestial Empire, and the sayings and doings of Commissioner Lin excited, at the time, no small degree of merriment. The time, however, is past, when either Her Majesty's Government or that of the East India Company would commit such an act of poltroonery as the following.

"The probable consequence of killing a Chinese would be this; that the Viceroy of Canton would, first of all, seize on the chief Supercargo, or, as he is here called, the 'Tipan;' and if he thought the business likely to prove very serious, perhaps all the English would be arrested; the man who committed the crime would then be demanded; for the Chinese have no idea of making a distinction between accidental and premeditated murder; as was fatally exemplified in the case of the poor gunner of an Indiaman some years ago, who was given up, because the wad of a gun, fired by the command of an officer, happened to strike a Chinaman in a boat at some distance, and occasion his death. It has never been known what became of the poor fellow; some have imagined that he was bow-stringed; while others think that his eyes were put out, and that he still lives an imprisoned victim to the narrow policy of the Chinese government."

We make a skip to Canton, of which we have a very lively account. We have imported much from Asia, and the "artful dodgers" of our metropolis are not so original as they may think.

"The streets of Canton are so narrow, and the concourse of people so great, that it is no very easy matter to make one's way through them in the day-time. These circumstances are indeed very favourable to a certain class of Chinese pick-pockets, who contrive to make out a livelihood by watching Europeans when they leave the factories, and following them until they see them in a throng of people, when they generally manage to pluck out their pocket-handkerchiefs, and sheer off with the booty. But this is not all: if they see an European of diminutive size, or seemingly weak, timid, or alone, and at any distance from the factories, three or four of these fellows will seize him in the middle of the street, and instantly rifle him of every thing he may happen to have about him at the time; the people in the shops tamely looking on, or perhaps applauding the rascals if they execute their manœuvres very adroitly! To a scene of this kind I was once an eye-witness; when another officer and myself prevented a gentleman of the g——s from being despoiled by these miscreants. They had seized upon him, pinioned him, and were on the point of stripping him, when we hove in sight, and forced them to abandon their intended prey."

It is to be hoped that an improvement has taken place in ship's crews since 1804.

"The following anecdote, related by a Captain of an Indiaman, and which, he said, happened under his own inspection, will give some idea of the manner in which the three days' leave is too commonly spent:—Among a party of sailors to whose turn it came to have leave for Canton, there was found one, who (in the sea phrase) had *bowsed up his jib* rather too much in the course of the morning. His messmates, however, handed him into the boat, and took him along with them to town. Here he plied the arrack bottle with such assiduity during his stay, that, in fact, at the expiration of his leave, the party brought him on board in full as good *sailing trim* as when he left the ship. A few days afterwards, when his intellects got a little clear, and the hands were turned up to move the ship to the second bar, the sailor went aft and complained to the Captain, that he had not yet had his turn of leave to Canton, peremptorily insisting, that, to the best of his recollection, he had not been over the ship's side since he left Gravesend! so complete a state of intoxication had he been in during his trip to Canton."

The stay at Canton was succeeded by a sail to Macao, where they passed a merry Christmas. On the 5th of January, 1805, with a stiff breeze that came down cold and bleak from the mountains, they gladly took leave of a country they had sought with such avidity a few months before—a not unusual sample of the "never is but always to be blest" principle of the human mind.

From China the fleet went to Prince of Wales's Island, where a severe attack of liver-complaint compelled Mr. Johnson to quit the ship and remain for some months. It afforded him an opportunity of seeing everything worth looking at, writing an agreeable account of what he saw, composing a little poetry after the manner of Darwin, and not only giving a sketch of the liver-complaint, but, what was better, getting rid of it.

In April, 1805, Mr. Johnson once more sailed for Madras, and bade adieu "to the pleasantest settlement in India—Prince of Wales's Island." He arrived at Madras Roads on the 21st of the month, to leave it on the 2nd of June, for the purpose of rejoining the Caroline at Vizagapatam. From many remarks upon many things, we select a few upon two subjects—sleeping in the open air, and the means of preserving health in India. For the first, under certain reservations, Mr. Johnson was an advocate. He thought it a wise and salutary plan to keep the awnings spread during the night, and to allow the men to sleep under them, especially in harbour, and in hot weather. This permission, however, he would withhold under the following circumstances :—

" First.—In the rainy season, or at the shifting of the monsoons, when the coolness of the air renders sleeping below a matter of little inconvenience.

" Secondly.—In those seasons of the year, when heavy dews fall during the night, and when awnings cannot be kept spread, to secure the crew from their baleful influence.

" Thirdly.—In rivers and other situations, where putrid exhalations are occasionally blown off from the swamps or low muddy shores, contiguous to the anchorage; and which should be guarded against, by sleeping below, and using large smoke sails."

Towards the preservation of health, he was of opinion that the practice of pouring cold water over the head every morning very materially contributes; and we cannot doubt that it does so in all climates. He approved (in hot countries) of frequently washing the lower decks—of paying great attention to the awnings both of boats and ships—of interdicting as much as possible labour in the sun—of a good but moderate diet—of wine in preference to arrack. He disapproved of permitting the men to go ashore —of sending them on expeditions in open boats—and of allowing them to attend funerals, a deadly office in the Tropics.

" In all cases where it is practicable, the ships which visit these unhealthy countries should anchor at as great a distance as possible from the shore; or if obliged to anchor near marshy grounds or swamps, especially during summer, or in hot weather, and when the wind blows directly from thence, the ports which would admit the noxious land-winds ought to be kept shut, especially at night.

" Or if the ship rides head to wind, a large smoke-sail should be hoisted forward, that the smoke from the galley, in ascending, might carry up and disperse the swampy shore effluvia.

" The best preservative against the mischievous impressions of a putrid fog, or of a marshy exhalation, is a close, sheltered, and covered place, in which there are no doors or windows facing the swamps. Persons on board any ship whatever are much more safe, and their situation is much preferable to that of those who make distant inland excursions in small boats upon the rivers, and who are for the most part ignorant of the cause of those maladies which destroy them. The intolerable heat at noon often obliges such persons to go in a manner half naked ; while a free and plentiful perspiration issues from every pore. A near approach to putrid swamps at this time, is apt to produce an immediate sickness and vomiting: and afterwards probably a fever. But if they happen to pass them at night, or lie near them in an open boat, the air from those swamps is perceived to be quite chill and cold; inasmuch as warm thick clothing becomes absolutely requisite, to guard the body against the impressions of so great an alteration in the air, and against its cold and inclement quality; for the effects of it then, even on the most healthy and vigorous constitution, is frequently a chilling cold fit of an ague, terminating in a fever, with delirium, bilious vomitings, a flux, or even death itself."

Appended to these observations we have the "Signs of an Unhealthy Country," which, whether coming from or sanctioned by one who had experience of so many countries, must be deemed not wide of the truth.

"1st. A sudden and great alteration in the air at sunset, from intolerable heat to chilling cold. This is perceived as soon as the sun is down; and is for the most part accompanied with a very heavy dew: it shows an unhealthy swampy soil, the nature of which is such, that no sooner the sunbeams are withdrawn, than the vapours emitted from it render the air damp, raw, and chilling, in the most sultry climates; so that even under the equator, in some unhealthy places, the night air is very cold to an European constitution.

"2d. Thick noisome fogs, chiefly at sunset, arising from the vallies, and particularly from mud, slime, or other impurities. In hot countries, the smell of these fogs may be compared to a new cleaned ditch. Diseases, therefore, arising from this cause, generally take place in the night, or before sunrise.

"3d. Numerous swarms of flies, gnats, and other insects, which attend stagnated air, and unhealthy places covered with wood.

"4th. When all butcher's meat soon corrupts, and in a few hours becomes full of maggots; when metals are quickly corroded on being exposed to the air; and when a corpse becomes intolerably offensive in less than six hours: these are proofs of a close, hot, and unwholesome country."

It cannot but be noticed, that those situations are among the most pestilential where heat by day is succeeded by cold damp fogs or exhalations at night. And in every quarter of the world, we apprehend that the concurrence of such circumstances is invariably prejudicial to the human frame. On the banks of the Hoogly it produces dysentery—on those of the Niger the deadly fever of Africa—in the fens of Lincolnshire it is redolent of agues—and by the side of the Thames it is fruitful of diarrhœa and rheumatism. The greater the contrast between the diurnal heat and nocturnal chill, the more pernicious the result, and although we would not be understood to deny that the more deadly consequences flow from specific miasms, we entertain no doubt that much, at the least, is due to the impression of damp and cold on the body, at once excited and exhausted by antecedent heat.

From June till October, 1805, Mr. Johnson was cruising on the Coromandel Coast, of which he gives us an account, together with one of the Island of Ceylon. The pearl-divers, of course, come in for notice. Accustomed to dive from their earliest infancy, these Indians fearlessly descend to the bottom in five or ten fathoms of water.

"A diving stone and net are connected to the boat by two ropes. The diver putting the toes of his right foot on the hair rope of the diving stone, and those of his left on the net, seizes the two cords with one hand, and shutting his nostrils with the other, plunges into the water. On reaching the bottom he hangs the net round his neck, and collects into it the pearl shells as fast as possible, during the time he finds himself able to remain under water, which is usually about two minutes. He then resumes his former posture, and making a signal by pulling the cords, he is immediately lifted into the boat. On emerging from the sea he discharges a quantity of water from his mouth and nose, and some discharge even blood; but this does not prevent them from diving again in their turn. When the first five divers come up, and are respiring, the other five are going down with the same stones."

The most skilful divers from the Malabar coast, are able to dive without the stones, and, for a reward, will remain under water for the space of seven minutes. So at least it is said.

In October, 1805, ill-health compelled Mr. Johnson to set sail again for Bengal, and on the 21st he reached the Ganges, at the time when Calcutta was plunged in gloom by the death of the Marquis Cornwallis. On the 3rd of November, at daylight, his Majesty's ship Medusa weighed anchor for England, and Mr. Johnson bade a final adieu to India. His feelings took the form of verse,—he modestly disclaims the title of poetry—recollections of the scenes he quits are merged in those of his youth—and the inspiration of his muse, averted from the burning clime of the East, points to his native skies.

Those skies were seen on the 26th of January, 1806, when the Medusa reached the Lizard, having run from the Ganges, a distance of thirteen thousand, eight hundred and thirty-one miles, in eighty-four days, two of which were spent at anchor in the roads of St. Helena:—perhaps the most rapid passage that has ever been accomplished.

The preceding account of the "Oriental Voyager" may seem disproportioned to its merits. But it comprises the history of three important years of Mr. Johnson's life; then it was he acquired his taste for varied and discursive information—tested his powers of description—made those observations on which he founded his work upon Tropical Climates—consolidated and confirmed his habits of industry—and, unfortunately, contracted the germs of a disease which saddened his future years, and doomed him to a late, though still a premature death.

His return from the East, somewhat shattered as he was in constitution, by dysentery, was a return to the dissecting and the lecture-room. The privateer and the Dutchman contributed their quota to the fees, and his prize-money enabled him to enter as a student at Guy's and St. Thomas's, where he formed the acquaintance and gained the esteem of Sir Astley Cooper, Dr. Curry, and the other ornaments of that celebrated school.

His professional education, so far as attendance on lectures went, was now completed. Desultory as it was, from the necessity of the case, we cannot but own that it was judicious in design and as perfect as circumstances admitted in its execution. Nor can we fail to admire the undaunted resolution of the man. Saving his few pounds that they might be spent on knowledge—returning when they were spent, to work and save again for the same object—and through the long period of eight or ten years, with every interruption, with those inducements to professional indolence that conquer and debauch the resolves of so many, with the obstacles (none know who have not felt them) of poverty and ill-health, having but one aim,—his education—and attaining it.

In the Autumn of 1806, he re-visited his native country, and became acquainted with Miss Charlotte Wolfenden, of a highly respectable family, in Lambeg, County of Antrim. This lady he shortly married, and she now survives him.

Soon after his marriage, he was engaged in attending the prisoners of war, in the depôts at Plymouth and Portsmouth. Here he made the most of his opportunities. In 1808, he was appointed to the "Valiant," of seventy-four guns, in which ship he remained nearly five years, was, we believe, in several engagements, and saw a great deal of active service.

The Valiant was one of the two line-of-battle ships that forced their way into Basque Roads, between strong batteries, and burnt the French fleet. His time in the Valiant was neither unprofitably nor unpleasantly spent. He saw much, made some friends, got, from the ship being the *worst* sailor in the squadron, a round sum of prize-money, and would often recur in after years to the stirring incidents of this period. It was in the Valiant that, in 1809, he was present at the disastrous expedition to Walcheren. He narrowly watched the progress of disease on those pestiferous shores, and his account of what he observed, in after conversations, was striking to the last degree. He did not observe only—he *felt*. He was attacked with the ague, and, although he threw it off tolerably well at the time, he was seized, like many others, years afterwards, and, without ostensible cause, with the same disease in a dangerous form, in London.

On quitting the Valiant he remained for a short time in the neighbourhood of Gosport. He was not idle. The notes that he had made in his voyage to India—notes that he had composed with diligence and preserved with care, were brought out, compared, corrected, and expanded into his principal work—*The Influence of Tropical Climates on European Constitutions.* But it is not enough to write a work—we must print it. This sine quâ non is frequently the difficulty, and perhaps many a production has missed immortality from the bad taste or prudence of a bookseller. This essential personage shook his head at the "Tropical Climates," and had not its author treasured up his prize-money for this very purpose, he would probably never have attained either reputation or a name. How subtle are the links that connect the chain of human fortune. If he had not saved from his pay to study, he would not have won the good opinion of Sir Gilbert Blane, nor been appointed to the Caroline—if he had not got the ship, he would not have enjoyed the opportunities in the East, of which he so decisively availed himself—and if he had not fallen in with a privateer, and husbanded his prize-money, those opportunities would have been, after all, profitless.

The publication of the work at once conferred on him a high character, and, immediately on its appearance, he was appointed flag-surgeon, with the late Sir William Young, then in command of the North Sea Fleet. He did the duty of physician to the fleet in a most inclement season, with constant hurricanes, and vessels foundering on all sides. So pleased was Sir William Young with his surgeon that he extended to him his patronage and friendship, to the last hour of his life. Sir William Young, indeed, was one of the two or three friends, whom alone he knew, and on whom he relied, when he afterwards embarked his all in the Metropolis.

The popularity and extensive sale of the work *On Tropical Climates,* render an elaborate notice of it needless. At the same time, some interest may attach to a few particulars respecting it, and a sketch of its main features may be pardoned.

The first edition was published in 1812, the sixth in 1841, after an interval of twenty-eight years. More than a quarter of a century had already set its seal upon its value, when that last edition was committed to the press. We may fairly presume that a work ushered in without pretension, established without patronage, and enduring amid the remarkable changes that have revolutionised medicine itself, must have been founded on observation, and contain some elements of truth. It is singular, indeed, how few of the author's original doctrines have been subverted by time, or practices condemned by subsequent experience, the more singular, as the work was substantially written when he was not twenty-four years of age. This is another instance to be added to the striking list adduced by Mr. D'Israeli of reputation atchieved in early life. That is, indeed, the period at which the energy is greatest, and, perhaps, the originality as well as boldness of the mind's conceptions most decided. In later years our judgment ripens, because our accumulated knowledge is increased, but caution begins rapidly to supersede that enthusiasm, which is mostly the source of the greatest actions.

One main feature in the work is its physiological basis. At its commencement the author considers, and in a great degree confutes, the notion, that the constitution of man is such as to enable him to defy changes of climate to a degree altogether unknown to other animals. In one sense this is true—in another, it is not. Man is endowed with those reasoning powers which give him in some sort the command of climates, as well as of the elements; but it is to those reasoning powers, rather than to his physical, that he owes this.

Mr. Johnson considers next the comparative advantages of stimulating drinks and water, in the East, and, in opposition to Dr. Currie, he proves theoretically, and asserts practically, the superiority of the latter. Not that he prohibits wine or spirits altogether, but, as a general rule, he maintains that they increase the perspiration, whose excess so greatly contributes to the exhaustion of the European in warm climates.

The sympathy between the skin and mucous membranes, a sympathy which we suppose that we explain, by looking on them as modifications of the same tissue, continuous one with the other, is adduced by Mr. Johnson as one reason for debility of the digestive organs.

"The loss of tone, then, in the extreme vessels of the surface, in consequence of excessive, or long-continued perspiration is, on this principle, necessarily accompanied, or soon succeeded by a consentaneous loss of tone in the stomach, and fairly accounts for that anorexia, or diminution of appetite, which we seldom fail to experience soon after entering the tropics, or, indeed, during hot weather in England. Now this, although but a link in the chain of effects, seems to me a most wise precaution of Nature, to lower and adapt the irritable, plethoric European constitution, to a burning climate, by guarding very effectually against the dangerous consequences of repletion. This view of the subject will set in a clear light, the pernicious effects of stimulating liquids, operating on an organ already debilitated (probably for salutary purposes), and goading it thereby to exertions beyond its natural power, producing a temporary plethora or excitement, with a great increase of subsequent atony."

The Liver plays a great part in the East. People there find out what an imperium in imperio they have in their abdomen. Bilious persons in temperate climates get a sick-headache, a diarrhœa in the Autumn, jaundice, perhaps, or a fit of gall-stones, but even they speak and think of their livers now and then only. In the East, Liver is the one thing considered—its derangements everybody looks for—it consigns to the early grave—it sends back its shattered owner to the shores of Europe before his time. To the Liver, then, Mr. Johnson devotes his attention, and its physiological actions and sympathies are the pivot on which much of his practice turns.

The first effect of a tropical climate on the organ is an increase of the biliary secretion. This is so indisputable, that it is enough to state the fact. Why should it do so? Mr. Johnson attempts to answer the question. We shall not introduce his facts and arguments in detail, but content ourselves with the general announcement of his views. Long as the quotation may be, it is valuable, and contains, in our opinion, the soundest physiological doctrine.

"There exists then between the extreme vessels of the vena portæ in the liver, and the extreme vessels on the surface of the body—in other words, between *biliary secretion and perspiration*, one of the strongest sympathies in the human frame, although entirely unnoticed hitherto, so far as I am acquainted. That these two functions are regularly, and to appearance, equally increased, or at least influenced, by *one* particular agent (atmospherical heat) from the cradle to the grave, from the pole to the equator, will be readily granted by every observer: and that this *synchronous action* alone, independent of any other original connexion, should soon grow up into a powerful sympathy, manifesting itself when *either* of these functions came under the influence of *other agents*, is a legitimate conclusion in theory, and what I hope to prove by a fair appeal to facts. This last consideration is the great practical one: for it is of little consequence whether this sympathy was originally implanted by the hand of Nature at our first formation, or sprung up gradually in the manner alluded to, provided we know that it actually exists, and that, by directing our operations towards any *one* of the functions in question, we can decisively influence the *other*. This is what I maintain; but here I only offer assertions. In a future part of the work I shall bring forward facts and cogent arguments in proof of them. At present let this 'consent of parts' between the skin and the liver, which I shall beg leave to denominate the '*Cutaneo-hepatic Sympathy*,' account for the augmented secretion of bile, which we observe on arriving in hot climates, corresponding to the increased cuticular discharge. I shall here offer one practical remark, resulting from this view of the subject, and which will be found deserving of every European's attention on his emigration to southern regions: namely, that as the state of the perspiratory process is a visible and pretty fair index to that of the biliary, so every precautionary measure, which keeps in check, or moderates the profusion of the *former* discharge, will invariably have the same effect on the *latter*, and thus tend to obviate the inconvenience, not to say the disorders, arising from redundancy of the hepatic secretion. To this rule I do not know a single exception; consequently its universal application can never lead astray in any instance. But this subject will be better elucidated, and more clearly explained hereafter.

To proceed. It is well known, without having recourse to Brunonian doctrines, that if any organ be stimulated to *inordinate* action, one of two things must in general ensue. If the cause applied be constant, and sufficient to keep up, for any length of time, this *inordinate* action, serious injury is likely to accrue to the organ itself, even so far as *structural* alteration. But if the cause be only temporary, or the force not in any great degree, then an occasional torpor, or exhaustion, as it were, of the organ takes place, during which period its *function* falls short of the natural range. To give a familiar example, of which too many of us are quite competent to judge:—thus if the stomach be goaded to immoderate exertion to-day, by a provocative variety of savoury dishes and stimulating liquors, we all know the atony which will succeed to-morrow, and how incapable it then will be of performing its accustomed office. It is the same with respect to the liver. After great excitement, by excessive heat, violent exercise in the sun, &c., a torpor succeeds, which will be more or less, according to the degree of previous excitement, and the length of time during which the stimulating causes have been habitually applied. For instance, when Europeans first arrive between the tropics, the degree of torpor bears so small a proportion to that of preceding excitement, in the liver, that it is scarcely noticed; particularly as the debilitated vessels in this organ, *continue* (similar to the perspiratory vessels on the surface) to secrete an imperfect fluid for some time *after* the exciting cause has ceased; hence the *increase* of the biliary secretion occupies our principal attention. But these torpid periods, however short at first, gradually and progressively increase, till at length they far exceed the periods of excitement; and then a *deficiency* of the biliary secretion becomes evident. This is not only consonant to experience, but to analogy. Thus, when a man first betakes himself to inebriety, the excitement occasioned by spirits, or wine, on the stomach and nervous system, far exceeds the subsequent atony, and we are astonished to see him go on for some time, without, apparently, suffering much detriment in his constitution. But the period of excitement is gradually curtailed, while that of atony increases, which soon forces him not only to augment the dose, but to repeat it oftener and oftener, till the organ and life are destroyed?

"Now it is somewhat singular, that this alternation of redundancy and deficiency, or, in other words, *irregular* secretion in the biliary organ, should pass unnoticed by writers on hot climates. They, one and all, represent the liver as a colossal apparatus, of the most Herculean power, that goes on for years, performing prodigies in the secreting way, without ever being exhausted for a moment, or falling *below* the range of ordinary action, till structural derangement, such as scirrhosity or tuberculation, incapacitates it for its duty! A very attentive observation of what passed in my own frame, and those of others, has led me to form a very different conclusion; and the foregoing statement will, I think, be found a true and natural representation of the case. I shall afterwards shew, that the secretion in question is frequently below *par*, in quantity, at the very time when it is considered to be redundant—all arising from irregularity and vitiation.

Here, then, we have two very opposite states of the liver and its functions. 1st. Inordinate action, with increased secretion—the periods gradually shortening. 2d. Torpor of the vessels in the liver, with deficient secretion—the periods progressively lengthening. In both cases, the bile itself is *vitiated.* We may readily enough conceive how this last comes to pass, by an analogical comparison with what takes place in the stomach during, and subsequent to, a debauch. In both instances, we may conclude, that the chyme passes through the pylorus into the duodenum, in a state less fit for chylification, than during a season of temperance and regularity. So, during the increased secretion, and subsequent inactivity in the liver, the bile passes out into the intestines deteriorated in quality, as well as superabundant or deficient in quantity.

" In what this vitiation consists, it is certainly not easy to say. In high degrees of it attendant on hurried secretions, both the colour and taste are surprisingly altered; since it occasionally assumes all the shades between a deep bottle green and jet black; possessing, at one time, an acidity that sets the teeth on edge; at other times, and indeed more frequently, an acrimony that seems absolutely to corrode the stomach and fauces, as it passes off by vomiting, and when directed downwards, can be compared to nothing more appropriate than the sensation which one would expect from boiling lead flowing through the intestines. Many a time have I experienced this, and many a time have my patients expressed themselves in similar language. The slightly disordered state of the hepatic functions, which we are now considering as primary effects of climate, and within the range of health, may be known by the following symptoms:—Irregularity in the bowels, with motions of various colours, and fetid, or insipid odour;—general languor of body and mind; slight nausea, especially in the mornings, when we attempt to brush our teeth; a yellowish fur about the back part of the tongue; unpleasant taste in the mouth on getting out of bed; a tinge in the eyes and complexion, from absorption of bile; the urine high coloured, and a slight irritation in passing it; the appetite impaired, and easily turned against fat or oily victuals,—irritability of temper—dejection of mind—loss of flesh—disturbed sleep. These are the first effects, then, of increased and irregular secretion of bile, and will appear in all degrees, according as we are less or more cautious in avoiding the numerous causes that give additional force to the influence of climate. For example: if I use more than ordinary exercise—expose myself to the heat of the sun—or drink stimulating liquids to-day, an increased and vitiated flow of bile takes place, and to-morrow produces either nausea and sickness at stomach, or a diarrhœa, with gripings and twitchings in the bowels. But a slight degree of inaction or torpor succeeding, both in the liver and intestines, there will probably be no alvine evacuation at all, the ensuing day, till a fresh flow of bile sets all in motion once more. These irregularities, although they may continue a long time without producing much inconvenience, especially if they be not aggravated by excesses, yet they should never be despised, since they, inevitably, though insensibly, pave the way for serious derangement in the biliary and digestive organs, especially in hot climates, unless counteracted by rigid temperance, and the prophylactic measures which I shall carefully detail in the proper place. The reciprocal influence and effects which the hepatic and mental functions exercise on each other, will form an interesting inquiry under the article Hepatitis."

Modern Chemistry has disclosed more facts with regard to the Biliary Secretion, which bear upon the subject. While it is indisputable that the bile acts as a sort of precipitant upon the chyme, throwing down, as it were, the excrementitious portion, and by its soda neutralising its acidity, it is equally clear that, charged as it is with carbonaceous compounds, it acts as subsidiary to the function of the lungs. Respiration is the more immediate agent in producing and maintaining animal heat, and modern physiologists believe that the act of respiration is less vigorous in high temperatures than in low ones. If this be so, if the lungs act less, in order that the carbon be eliminated, the liver should, or, at all events, *might* act more: another reason why the liver will be stimulated in hot climates. But as that same heat stimulates the skin also, we see at once how it happens that a sympathy is evinced between the two organs. In short, the lungs and the skin are associated in the maintenance and regulation of animal temperature—the lungs and the liver are associated in the separation of the carbon—and, perhaps, less obviously, the liver and the skin are associated in the regulation of the temperature too. These physiological considerations founded on data furnished by chemistry and by experiments many years after Mr. Johnson wrote, amply confirm the accuracy of his observations, and strengthen the practical value of his inferences.

Mr. Johnson passes from these preliminary views to consider Fever in General, which leads him to the Endemic Fever of Bengal. After this he takes up in succession the Endemic of Batavia—the diseases of the Mediterranean—the fever of the Coast of Africa—and the maladies more peculiar to the Western Hemisphere.

In enumerating these as the contents of the work we are rather referring to the later editions than the first, which is not just now within our reach. As this memoir does not profess to be purely and critically bibliographical, the taking one edition as a sample of all may probably be pardoned. It is enough to observe, that the portions of the work founded upon his own observation, are scrupulously distinguished from those for which he had to draw upon others. The many quarters of the globe, with the still more numerous diseases incidental to them, that form the subjects of the volume, preclude, of course, the notion that one man or one lifetime could individually grapple with them. But the leading idea, the general spirit that pervades the book, is exclusively Dr. Johnson's, and the aid of others is invoked to illustrate and to expand, what one mind could not produce in its actual form, and completeness, and circumstantiality.

It would be a hopeless, and an useless task, to endeavour to present in any such compass as we must be restricted to, an analysis of the work on Tropical Climates. A physiological as well as a practical spirit pervades it, and, we see in it the leading characteristics of the author's mind —clearness of observation—paucity of theory—simplicity of precept—decision of action—and, conjoined with a vigorous independence of thought, that considerate deference to the capacity of others, which, while it marks humility, implies candour and judgment, and is often consistent, in philosophical inquiries, with great strength of understanding. Dogmatism, in fact, is rarely seen, but in those of small experience, or of narrow views.

Nearly six thousand copies of this standard work have been distributed throughout the Globe. It is found where streams the pendent of a British man-of-war, or waves the flag of our commercial marine. It is a text-book in the old world and the new, in the Provinces of China and in the West Indian Islands. The sun never sets upon its page, and on its doctrines the lives of countless thousands have been ventured, and of numbers are, this moment, staked.

The best and most complete edition is undoubtedly the last. In the arrangement of that, Dr. Johnson had the valuable aid of Mr. Martin, formerly Presidency Surgeon in Bengal, and Surgeon to the Native Hospital of Calcutta. Mr. Martin informs us in the part of the Preface assigned to him :—

" The materials of the brief sketches now incorporated in Dr. Johnson's well-known work, appeared originally in an official report on the climate and diseases of Calcutta, of which two editions were ordered to be printed at the public cost by the Supreme Government of India.

"These sketches were the result of observations made in the earlier period of my service, with troops engaged in active military operations in some of the most unhealthy countries in India, and in the Burman Empire, during the late war with that State; confirmed subsequently in an extensive hospital and private practice amongst both Europeans and Natives, in the British Indian Capital. The mode of their first appearance was, as I have said, official;—indeed, they were thrown together without care, and in the hurry of excessive professional labour, with the sole view of affording such facts and materials as might assist a public inquiry I then suggested into the health history of Calcutta, and the existing condition of that city and the surrounding country, with a view to their improvement; as I had very early satisfied myself that, if we would effectually attend to the welfare of its vast population, we must adopt measures of correction and prevention in vigorous and systematic form. On this subject, I had previous official and personal communication, first in 1821, and then in 1828, with the Governors-General of India; but it was not till 1835 that effectual measures were taken for a full investigation into the various and important matters connected with the subject."

Of Mr. Martin's contributions it is impossible to speak too highly. The result of two and twenty years' observation in India, we may well suppose they are derived from a varied and vast experience. But it is not experience only they evince. Those who have the pleasure of Mr. Martin's acquaintance can speak to his independent and cultivated mind, his clear views, his patient investigation, and his sound judgment. The portions that he has written are indicative of all these qualities, and are at once lucid and complete analyses of the subjects they embrace. His Sketch of the Climate of Calcutta is one of the most masterly things of the kind.

The manner in which Mr. Martin came to be connected with Dr. Johnson, in this last edition, is flattering to the one and characteristic of the other. Mr. Martin, who was entirely unknown to Dr. Johnson, consulted him on the state of his health, enfeebled by his long residence in Bengal. Dr. Johnson gave the best advice in his power, and was so impressed with a high value of Mr. Martin's opportunities and talents, that he frankly offered to associate him with himself in the editing of an Edition then in the press, and proved a most warm and liberal friend in furthering his professional views.

At the peace of 1814, King William the Fourth, then Duke of Clarence, hoisted his flag in the "Impregnable," 98 gun ship, when Sir William Young retired. Mr. Johnson was so strongly recommended to the Duke, that he was retained, and served with his Royal Highness, while conveying the Emperor of Russia, King of Prussia, and other Potentates to England. The Duke had an attack of his hay-asthma at Boulogne, Mr. Johnson attended him, and the attack was speedily subdued. This and the frank and simple character of his surgeon, so congenial to his own, pleased the Duke highly, and was the foundation of what might almost be called a friendship. At the time, this was shewn by the Duke's exerting all his influence to obtain for Mr. Johnson the rank of physician to the fleet. But the King's son was baffled by the the First Lord of the Admiralty, and Lord Melville negatived the application.

The Duke of Clarence then, and to the time of his accession, had little personal or political influence. With narrow means, a large family, and a remote chance of the Crown, he could give little to his friends but empty titles and good wishes. To Mr. Johnson he extended both. He appointed him his Surgeon in Ordinary, and ever expressed a warm interest in his welfare. The letters, of which some from the Duke still remain in the hands of Dr. Johnson's family, are rather those of private friends in ordinary life, than such as might be supposed to pass between the Heir Apparent to the English Throne and a subject without rank or influence. As most of the letters refer to events of a private nature, and to persons who might not wish their names brought forward, we shall content ourselves with quoting one on a more public subject,—the death of the Duke of York. Dr. Johnson had written to the Duke of Clarence condoling with him on his loss. The Duke thus answers his letter :—

Bushy House,
*Jan.* 31*st.*, 1827. *Late at Night.*

Dear Sir,

I am to thank you for your second edition, and your letter of 29th instant. I feel very sensibly your kind interest on account of the mournful event of the death of the ever to be lamented Duke of York. I agree with you entirely that things of this kind teach mankind the will of God and the necessary submission of mortals to the power of the Almighty. How transient power, riches, and even happiness itself—the mighty preparations for the Coronation gone in one day, and the death, though long expected, of my poor brother, are lessons I cannot forget.

I know your esteem for me, and am therefore grateful for your very proper and religious remarks. I am, thank God, in good health, and trust you are equally so. Adieu, and ever believe me,

Dear Sir,

Your's sincerely,

William.

It might have been expected that William the Fourth would not forget the friends of the Duke of Clarence. But Kings are surrounded by an atmosphere which is rarely pierced by the simple light of services, and which is too brilliant with the present to exhibit the fading past. At the time when William the Fourth began to reign, one individual decreed the medical appointments of the Palace, assumed a sort of vested right to head and make them, and no man, while he retained his influence and post, could hope, without his patronage, to be made Physician to the King. The list was published, and Dr. Johnson's name omitted. This was "too bad." He wrote himself to His Majesty, and appealed to him to say, whether it was by *his* command, he was excluded. The answer was significant. He was made Physician Extraordinary to the King, in despite of the traditions of College and Court, and of all that back-stairs influence, so readily imagined, so dirty, and so persevering. Monarch and Physician have both passed away —their spirits stand before an awful, and a just tribunal—the idle distinctions, sought or conferred, must be to both an unheeded folly—but if there be a remembrance of the past, who will not say that that remembrance will be saddest, with the Prince who yielded to a paltry clique, and deserted, in some degree, his friend! But let us not do an injustice to one of the kindest and honestest of monarchs and of men. No one, in his rank, has ever displayed less of the selfishness too characteristic of our aristocracy, or been at less pains to repress or conceal, in obedience to the hollow heartlessness of class, those generous impulses which stamp the individual a member of the family of man. The

Nihil humanum à mæ alienum puto was the practical creed of a Prince, who walked up Piccadilly with an umbrella under his arm, immediately after his accession, and retained the simplicity and single-mindedness of the sailor at the expense of the reserve, and even of some of the dignity of the Sovereign. In the present case, his feelings were displayed in violation of prescriptive forms, and, probably, to the astonishment, perhaps to the horror, of those courtiers who had been accustomed to the polished callousness of George the Fourth. When Dr. Johnson was presented at the Drawing Room, after his appointment, King William took his hand and shook it heartily, addressing to him at the same time some good-natured words of recognition and welcome. This act of unexpected condescension took Dr. Johnson so much by surprise, that he imagined *he* must have committed some flagrant act of indecorum, and wrote to apologise for it. The reply, as might have been expected, set the matter in its proper light, and was characteristic of the King.

It may seem that too much has been said, and that the appointment was of too insignificant a kind, to be made a subject of congratulation or regret. Dr. Johnson was not the man to set a high value on the highest honour attainable by professional exertion. His own simplicity of character underrated rather than overrated the distinctions that so many covet, and he expressed and evinced indifference, almost contempt, for the insignia of wealth and rank. But the Duke of Clarence had been a personal friend, to exclude him from the King's medical staff would have been a personal slight, he knew that King too well to believe it could be done by him, and he felt the indignation which every man of spirit must feel, at being made the victim of intrigue. He was too independent to become, or wish to be, a hanger-on at Court, and he left to those who liked them, its suffocating atmosphere and gilded chains. William the Fourth was soon gathered to his fathers. Another reign scouted the man who had so long monopolised and still grasped at power; and, overtaken by neglect, if not by contumely, he crowned an unhonoured age by an unrespected memory.

We have been obliged to anticipate, and we return to events in their chronological order.

When the long war ended in 1814, Mr. Johnson felt that his naval life was run, and that the future must be sought and found on shore. His desire and intention were to practise as a Physician,* for which he was best qualified, both by his general and professional attainments. But to succeed as a Physician he must first be known, to be known must be a work of at least a few years, and during those years of preparation and of trial, himself and his family must live. His resolution was shortly taken, and it proved, as it seemed like to be, a sound and a successful one. That resolution was to address himself at once to Medical Periodical Literature, to build upon it a name, an income, and the power of practising in the capacity he had selected, while, in the interim, he should draw support for those dependent on him, from his half-pay as a naval surgeon, and general practice among his old comrades and brother-officers in a sea-port town. He naturally selected Portsmouth, where he speedily became well known, and

---

* He had taken out a Scotch degree in the preceding year.

realized for the brief time that he remained and the limited field open to him a very remunerative income.

While serving with Sir William Young, in the North Sea, he had published some papers in the "*New Medical and Physical Journal.*" Of this he now became the Editor, in conjunction with Drs. Shearman and Palmer. In 1816, it appeared under the title of the Medico-Chirurgical Journal, in a monthly form, comprising about 100 pages, and divided into four parts, which were devoted to Original Communications,—to a comprehensive Analytical Review of Medical Literature,—to Selections, principally from the Foreign Journals,—and to Medical Miscellanies. The Journal itself met with no great success, so far as money went, but it answered Mr. Johnson's purpose. Though nominally one of three Editors, he had had, and that by choice, the lion's share of the work—he had tried and skilled his prentice-hand on criticism—he had made himself a name which, although not great, was still a name—he had accumulated and was accumulating knowledge—he had proved to himself that he could win the confidence of patients, and that the rough frankness of the sea could soften into the milder requirements of the shore—he had sustained himself the while—and now the time was come for doing what he had resolved to do, and what all that had been done was intended only as the means to help him to.

In 1818, he bade adieu to Portsmouth, and took a house in the Albany Court-yard, in London, becoming a Licentiate of the College of Physicians in 1820. He had indifferent health—one friend in the metropolis, Sir William Young—a wife and five children—something less than five hundred pounds—and his half-pay, under a hundred a year. The risk was palpable, ruin might seem, even to a sanguine vision, looming in the distance, but what are these to an undaunted will, and a consciousness of power? Like Sheridan he felt, when his first speech failed, that—"Damn it, it was in him, and it *should* come out," and like Sheridan he found the conviction the means of its own realization.

We have said that he took a house in the Albany. He took at the same instant a bolder step—he changed the Monthly Medico-Chirurgical Journal into the Medico-Chirurgical Review, and published it thenceforth as a Quarterly Journal, at his own sole risk and expense. He was prompted by his courageous spirit—necessity—the conviction that a good analytical Review ought to emanate from London—and the ambition to prove, in his own person, that that conviction was a true one. What visions of reputation and of profit dimly appeared in the future, we may conjecture, but we cannot tell.

Of the progress and the fortunes of that Review we shall give a brief account, while for a time we desert his own.

The Review was still conducted on the same plan, but by degrees it might be observed, that the portion devoted to Original Communications became diminished, whilst that reserved for analysis of works became extended in proportion. Though this, of course, increased greatly the labour of the Editor, (it being far more easy for him to insert an original report of any case that might be sent to him, than to sit down and condense the pith of some long-winded work into as many pages as there were volumes in the original,) it was persevered in, and met with signal success. The number of subscribers augmented rapidly, and, though a greater quantum of *concentrated matter* could be seen in no equal number of pages in any medical work in Europe, the ardour which at that time (1819) pervaded the world of medical science, the number of new and important publications daily issuing from the public press, and the rapidly increasing list of correspondents, rendered it necessary to enlarge the limits of the Journal. The Editor considered that the British Metropolis should send forth a respectable Quarterly Register, inferior to none; and accordingly exerted himself to the utmost to place his own in that position.

Success attended his efforts;—his health, which had been for some time greatly impaired, improved;—and there began to appear some prospects of professional success in the sphere which he had lately selected. At the termination of the first year of the Quarterly Series he was enabled to write as follows.

"The first volume of the Medico-Chirurgical Journal has closed, under an expression of public approbation, unprecedented in the annals of Medical Literature. This approbation, while it affords the Editor an honourable source of independence, as well as gratification, insures a constant stimulus to increased exertion. A renovating health, a redoubling zeal, a spreading circle of correspondence, and a widening sphere of observation, shall all be combined—with indefatigable industry—to render this Journal worthy of the flattering patronage with which it has been already honoured by a generous and liberal public. To say more would be unnecessary—to say less would be ungrateful. It is by performances, not promises, that the Editor hopes to maintain the confidence of his professional brethren, and contribute his mite to the diffusion of Medical Science."

In 1820 the Review experienced a further modification. If we trace the History of Arts, Sciences, and every Human Institution, we perceive an endless series of changes and innovations marking their progressive advances towards perfection or maturity. It is, in truth, the province of wisdom and philosophy to profit by experience, and take each day a lesson from the past; while it is the character of folly and arrogance to vacillate without cause, change without object, or persevere against conviction. The "tenax propositi" is a dangerous maxim in Literature, as well as in Politics. The changes, then, which this Journal underwent, are not to be ascribed to caprice or instability. It will be found, on examination, that each innovation was an improvement on the preceding form, and an addition of labour and responsibility to the conductors.

It was found impracticable to keep pace with the progress of medical science in the *Review Department,* and at the same time allot, in every number, a sufficient space to Original Communications and Foreign Translations. As testimonials were received, from every quarter, of the most unequivocal approbation of the *Critical Analyses,* made by Dr. Johnson himself, with a closeness and precision beyond all precedent, and solicitations to carry them to the utmost extent, it was determined that the Medico-Chirurgical Journal should assume the character of an *Analytical Review.*

The paramount objects of the work were stated at the time by Dr. Johnson to be:—

First.—To generate in the minds of medical students an early turn for observation; a habit of reflection; and a taste for medical literature.

Secondly.—To bring within the reach of those classes of medical society who may not have the leisure to peruse, the means to purchase, or the opportunity to obtain, the various works that issue from the press, a *comprehensive* yet *concentrated analysis* of all important medical publications enlivened with practical rather than biblical criticism, which last is more calculated to indulge the vanity of the writer, than improve the understanding of the reader.

Thirdly.—To enable those who *have* the means and the desire of forming a selection from the current of medical literature, to judge for themselves, without the previous labour and expense of perusing and purchasing many *useless* publications.

It must be admitted that an *Analytical Review,* on a large scale, is highly valuable, not only for the purpose of reflecting the great features of medical science diffusedly through the profession, but also of fixing them in a form of record conservative of what is most valuable, and calculated alike for present perusal and subsequent reference. The latter object was kept steadfastly in view, from a thorough conviction that, by declining all temporary matter, and registering that only which would be read with interest, and referred to with advantage, a work would be constructed, the volumes of which would become more valuable in proportion to their age. What an inestimable treasure would now be an Analytical Review of all that has been published during the last hundred years! and with what interest can we now look back upon the labours of our author, concentrating in a single work the medical science of an era, distinguished as it was by emancipation from prejudice, and by a rapid advance and general movement not often met with in the world of medicine.

But whilst the medical readers at large were benefited, medical writers derived no small advantages. To have their works analytically portrayed before the whole professional circle, leaving the judgment in general to the public itself, is of no mean service to authors, stimulating the deserving to increased exertions, and allowing those exertions to be brought home to the profession at large.

That the Medico-Chirurgical Review executed the task undertaken with credit to itself and advantage to the public, was soon shown. It became necessary again to enlarge the numbers, nearly to their present size, and not only was its reputation and consequent sale, widely spread at home, but its fame extended also throughout Europe, and across the Atlantic.

The eclectic or leading articles, the supplemental reviews, and all the principal analyses became regularly republished in America; while extracts from many of them were diffused through the continental periodicals. It could not but be gratifying to authors to know that *full accounts* of their productions were read not only wherever the Journal travelled, but that, by branching into new channels, in various countries, they continued to be circulated to an almost inconceivable extent. In reciprocity of intellectual intercourse this work collected into a focus the prominent rays of science and useful knowledge, beaming from other and distant regions, thus alternately diffusing light by reflection, and receiving it in return.

"Un immense reseau," says an eloquent foreign medical writer, "de communication entre tous les savans de l'Europe, et meme du nouveau monde, entretient l'eveil des esprits, repand les decouvertes comme l'éclair qui brille soudain à tous les yeux, etablit cette harmonie de sensibilité morale entre toutes les âmes éclairées, et les fait participer en meme temps aux pensées les unes des autres."

In 1824 it was determined to commence a new series. The reason for this was, that the back numbers were out of print, and few people like to begin with a broken set of a periodical journal. The scope and tendency of the Review were now well known; the plan adopted, viz. that of discarding all original articles, and confining the work to analytical reviews of books, and selections from the various Journals, time had proved to be not only perfectly practicable, but highly satisfactory. While, unencumbered with original communications, the Periscope enabled the Editor to select all the really valuable novelties that appeared. It was true that they were had only at second hand, but this in many respects proved an advantage. If the articles would not bear keeping for three or even six months they were hardly worthy of appearing in the Review at all. There is, in fact, great variety in these commodities. Some keep a week, and no more. The moment they see the light they perish. Others keep a month and then languish. If they stand three months, they are worth a trial, and the best specimens of these appeared in the Journal, mixed with the best samples from abroad.

From this time the Medico-Chirurgical Review enjoyed a quiet and tranquil existence; its popularity was fully established; its plan finally settled. In 1834, Mr. Henry James Johnson, who had then but lately served the offices of House-Surgeon to St. George's and the Lock Hospitals, became associated with Dr. Johnson in the management of the Journal, having long, however, written a large portion of it. In 1843, Mr. Johnson, who had then become Lecturer on Anatomy, and also Assistant-Surgeon to St. George's Hospital, found himself unable to devote the proper amount of time to the Review, and accordingly removed his name from the title-page, though he continued to write numerous articles.

In October, 1844, Dr. Johnson, finding that his health and strength were no longer sufficient to enable him to support the constant fag of the Editorship of the Review, in addition to the toils of his extensive practice, most reluctantly determined to resign its management. We are convinced that it was a much more severe pang for him to do so than would readily be imagined. For nearly thirty years had he been engaged with it—he looked on it as a creation, a child of his own—they had started together in this vast metropolis, unknown and friendless—together they had thrived—and now, when success had been attained, it was a hard blow for him to resign it to the care of others.

The deed, however, was done; and the Medico-Chirurgical Review passed into the hands of its present proprietor. Dr. Johnson from that time ceased to have any direct interest in it, but he had the consolation of feeling that no trouble, no expense would be spared by the new management in their endeavours to deserve the continuance of that amount of patronage which in his hands it had obtained.

The preceding historical sketch of the Medico-Chirurgical Review will not be uninteresting to *our* readers at all events. Probably it will not be so to any who peruse the life of Dr. Johnson. To start it, to conduct it, to resign it, were all epochs in that life, connected, by the subtle thread of destiny, with his best hopes and aspirations, his most highly-prized successes, and his bodily decline.

We have already said that to settle in the Capital, and to publish the Journal were simultaneous events. It was on that Journal he relied, to procure for him a position in London, practice, reputation, everything. A man who could make so bold a venture would not be likely to flinch in the conducting of it. Nor did he. Early tastes, a lively fancy, an enthusiastic temperament, the prize in front, the abyss behind, stimulated to the utmost his natural industry. So ready was his pen, that he rarely or never read the copy of his articles before they went to press—so accurate, that the cost of corrections after their return seldom exceeded a few shillings a quarter—so easy and so vigorous, that never has there been a Journal less infected with dulness. Yet facility of composition was, in his case, the reverse of copiousness of words, for terseness and conciseness stamped every line.

But, whatever his literary powers, a work of the magnitude of the Medico-Chirurgical Review, a work, be it recollected, essentially analytical, could not have been conducted as he conducted it, for thirty years, without a quality that has been found in the greatest men, and seems essential to the accomplishment of the greatest actions. That quality is *punctuality*. It was Dr. Johnson's motto and his practice—it entered into everything that he did, little or great, bodily or mental. In one sense, indeed, he was *not* punctual, for he was always rather before his time than after it. He calculated the pages which must be written daily to secure the due publication of the Journal. That number he made it a point of conscience to write, before he retired to bed. Weary or not, ailing or weak, in or out of spirits, what was to be writ was writ. If practice did not press, and he had leisure time, he anticipated the future, and wrote far in advance. His tours of health, which he commenced early and continued almost to the last, were fairly won by work before hand. He never set out without leaving the Journal in readiness, so far as writing went, for the next quarter-day. For ten or twelve years almost every article was written by himself, a circumstance, we imagine, unexampled in periodical literature.

His Tours of Health were tours of toil. Before setting out on them, every book that referred to them was read, the maps studied, the route chalked out, the posts arranged. Before he saw a place, he knew more of it than many who were born and bred in it. No sooner was the carriage at the hostel, than Dr. Johnson was not to be found. You looked in vain for him in-doors. He might be in the grandest street, in the dirtiest alley, inside the church, outside the theatre, but the most likely place to find him was on the top of the very highest hill in the neighbourhood. For this he always made at once, effected a sort of general reconnoissance, and studied details afterwards. Prepared before-hand, he knew what to look for; and that is the secret, in touring, as well as in many other things, of making the most of time. Whilst there was light he was ever on the move, and, like the Athenian, his motto seemed to be, action, action, action. Darkness alone drove him in-doors, to the light supper—but not to the early bed. What he had seen he would remember, and what he would remember he knew well, from early habits and long experience, he must note. The objects of the day, and the impressions of the moment, were recorded, in language fresh as the images that prompted it, before the tourist, however knocked up by the hills he had climbed, or the weather he had braved, consented to seek his couch. This was the method by which he saw so much in so short a time, and described with such spirit what he saw. In pleasure, as in business, (for pleasure was business,) we see the same natural aptitude for indefatigable exertion—the same love of order, regularity, and method.

His industry, indeed, was not only indomitable, but it can have rarely been surpassed. He conducted the Journal, built up an extensive private practice, read all that was worth reading in medical, not a little in classical, and extensively in general literature, composed, at short intervals, a series of popular works of by no means inconsiderable bulk, revised new editions of former ones, and took his annual tours of two or three months' duration. To effect this, there must be natural ability as well as industry, the power to do a thing quickly and well, with the resolution to do it punctually.

Dr. Johnson had determined that the Journal should be the means to practice, and it proved so. While it gave him a present income, it gave him also a name, and a name is the passport to professional success.

Patients came to him from the country, from the Continent, from the East and West Indies, from America; and though men, with peculiar opportunities, have carved out a reputation of a higher order, and amassed much more wealth, it will probably be long before a physician will, without those aids that do so much for the ablest and most fortunate, obtain consulting practice to the same extent, and drawn from so wide a range.

An excellent constitution had been shaken by fever in India and at Walcheren—was shaken now by this incessant toil, and, we may well suppose, anxiety. In the Winter of 1822, hæmorrhoids and prolapsus ani, from which he had suffered since his dysenteric attacks, led to the formation of matter in the cellular membrane round the rectum. Two operations, of some severity and risk, were requisite; Sir Astley Cooper performing one, Mr. Guthrie the other. Both these gentlemen were his personal friends, and shewed him the most considerate kindness. He recovered after a long and painful illness, and shortly afterwards removed from Spring Gardens, where he had lived for the last few years, to 8, Suffolk Place, Pall Mall, where he afterwards resided. But his nerves were unstrung—his digestion never good, was impaired—the most gloomy images continually haunted him—and, to such a pitch did the mental depression consequent on dyspepsia reach, that, in the midst of the London season, with his practice curtailed by previous illness, he broke up in despair, and retired to Margate, as he thought, to die. The keen breezes of the Foreland restored the tone of the stomach, and with the indigestion went the gloomy and desponding ideas that it had generated. He returned to town in the course of a few weeks, with a body refreshed, and a mind as resolute as ever. How mysterious are the links in that chain of events, which drags us to our good or evil destiny. The calamity of to-day proves our salvation to-morrow, and what seemed the happiest stroke of fate, is the parent or the harbinger of ruin. So Dr. Johnson found it. The complaint which prostrated body and soul, and from which he predicted nothing but beggary, was to make his fortune. His own sufferings directed his attention more particularly to dyspepsia, and were the foundation of his Essay on Indigestion, which was drawn most vividly and truly from the life.

This Work, which bore the following Title;—*An Essay on Indigestion; or Morbid Sensibility of the Stomach and Bowels, as the Proximate Cause, or Characteristic Condition of Dyspepsia,* was published in 1826. Its success was the most striking thing imaginable. It took the town, at least the dyspeptic part of it, by storm. Every victim to indigestion bought or borrowed it—he found in it his own sensations described, his own miseries painted by a master-hand. It went through four editions in little more than nine months, and has reached and nearly exhausted the tenth. It was found on almost every table—it was reprinted in America—it was translated in Germany and France—it received at once the sanction of public and professional approbation—and it raised his practice, with magical rapidity, to the highest point consistent with his health, and his very moderate desires.

When we examine the work, to ascertain the cause of this excessive popularity, we are struck with its conciseness, clearness, simplicity, and truth. It describes, in plain and easy language, just what the author felt and saw. Its precepts are judicious, its practice simple. This was the secret of its vast success.

Of the next three years of his life there is little or nothing to be said. They were devoted to those avocations, which left few minutes of a long day unoccupied. In the Autumn of 1829, he made a lengthened and agreeable excursion to France, Switzerland, and Italy. He had previously made a not very dissimilar tour in 1823. The impressions of the first were strengthened or corrected by the last, and the result was a work which saw the light in 1831. It was intituled *Change of Air, or the Pursuit of Health and Recreation.*

To this work he was partial, and erroneously, in our opinion, looked upon it as his best. No doubt, it is written with spirit, and displays the bent of Dr. Johnson's mind, which, like his reading, was various and discursive. It records too the pleasantest hours of his life, when he travelled for amusement, novelty in every scene, health upon every breeze, and care no longer recalling the past, to dash the enjoyment of the present, and obtrude shadows of the future.

The first subject that occupies his attention is that striking effect of anxious and stimulating occupations, more visible in England than in any other country, and most of all marked in London. It is the "wear and tear" of the body from intense exertion of the mind, that exertion rendered so necessary by the mere desire to keep our place, to say nothing of the ambition to improve it. The pale face, the lined features, the drawn angle of the mouth and nose, the dingy complexion, and, very commonly, the impaired digestion, are the characteristic symptoms. This disorder, for disorder it is, Dr. Johnson sketches with a master's hand, and for this he proclaims change of air and scene the antidote. And no doubt it is so. There are few in this great city who have not experienced the complaint, who have not found the value of the remedy. A high civilization brings its evils, but it brings some benefits too. The enormous increase of our manufacturing towns would bury their populations in their dingy streets, and murky atmospheres, did not that manufacturing wealth, which springs from them, cover the face of the country with railroads, plough our rivers and seas with steam-boats, and decorate our suburbs with villas. The wealthy man retires to these after his day of toil, while the petty tradesman and the poor mechanic are enabled, for the most trivial sum imaginable, to escape, on one day of the week at all events, from the unceasing labour of the six.

The "Change of Air" is not exactly a guide-book, nor is it a mere tissue of reflections. It is both, and probably a more amusing and companionable work could not be taken on the route its author traversed. Switzerland, Rome, Naples, Pompeii, Pisa, Genoa, Nice, pass in review before us, and we are continually met by some shrewd and original view of things and places we had thought familiar. No conventionalities imposed laws on Dr. Johnson, and if he thought a rock bleak he said so, the whole band of connoisseurs and dilettanti to the contrary. This independence of thought and expression produced its good effects, and in the case of the relics of Pompeii, was the means of leading the authorities of Naples to a juster sense of morality and decency. Indeed, we could not well refer to a chapter more characteristic of Dr. Johnson as a writer, than that very one. Rough, ready, humorous, caustic, and shrewd, a common-sense, Englishman-like view is taken of a Campanian city, and no falling in with fashionable cant, no affected admiration of antiquity, makes him hesitate to stigmatise, as a good man should, the abominations disclosed. His sketch wants the high polish and exquisite taste of Sir Lytton Bulwer's novel, but we have reason to believe that the latter acknowledged some little obligation to the author of "Change of Air."

The last Tour he made was in his native country—the last work he wrote was upon Ireland. The same bodily vigour characterised his journey through the Emerald Isle—the same sprightliness of description was found in his account of what he saw. In this Irish Tour, will be found also that independence of mind, which made him cry

"A plague o' both your houses,"

and rendered him alike indifferent to the shibboleth of every faction, Tory, Radical, or Whig. The wretched condition of the peasantry powerfully excited both his pity and his indignation, and he did not scruple to reprobate in the strongest terms, the mismanagement and misgovernment, social and political, which have afflicted Ireland, which have changed her labourers to paupers and assassins, her landowners to tyrants or absentees, curtailed her scanty manufactures, and made the English capitalist avoid her shores as he would a robber's den. Present at the great Tara meeting, Dr. Johnson had an opportunity of looking Repeal and Repealers in the face, and his natural good sense was far too robust not to make him penetrate, with sorrow and contempt, that enormous humbug. He expressed his sentiments on the subject freely, and even those who quarrelled with their freedom could not but admit their honesty.

We have said that this was his last tour and the last work of Dr. Johnson's. He contented himself thenceforward with the duties of his private practice, and amused himself with general literature. Scarce a work of any character appeared, but he perused it, and this constituted his recreation after the labours of the day.

He was now advanced in life. At the age of 67, he still carried his years bravely,—his hair was scarcely bleached by so many Winters—it had grown a little, and but a little, sparse upon the crown—his frame was still erect—his step firm—and not a tooth was missing from its appointed place. To this he would frequently refer with satisfaction, and boast that he had never had a tooth-ache. He sent his patients to the dentist, but he never went himself. The only thing which his family and friends observed, indicative of the effects of time, was a gradual but perceptible attenuation.

Perhaps a slight sketch of the man, as he appeared about the prime of life, may not be considered impertinent. It brings the subject of the memoir more palpably before us.

He was rather under than above the middle height, spare though of an active make, with a ruddy complexion, remarkably large and intelligent eye, bushy eyebrows, square and copious forehead, and an expression in which unmistakeable benevolence was shaded with a cast of care or melancholy, it was not easy to say which. In conversation, the features lost this character completely, and assumed what you would suppose were alone natural to them, that of unalloyed cheerfulness. Plain in dress, though never slovenly, simple in his manners, unaffected in every thing, he communicated the idea of being just what he was, and of not wishing to be taken for anything else. For affectation, indeed, he had a supreme contempt, and pretension, of whatever character, or in any quarter, felt the power of his tongue and pen.

His outward was, as much as it was possible to be, an index to his inner man. A disposition of unmitigated benevolence and kindliness was cloaked, in some measure, by that testiness of humour which so constantly conceals great goodness of heart. It is hard to explain this apparent inconsistency, but it is a common natural phenomenon. A rough word was sure to be succeeded by some substantial kindness, and so well was this known that it was played on.

Many who are not born to fortune, and acquire money by their own exertions, display a love of it, which lapses into avarice or parsimony. Such was not the case with Dr. Johnson. Liberality was a prominent feature of his character, and was stamped in every thought and act. It was not merely that he did not amass wealth with greediness, and hoard it with tenacity—he was liberal in money-matters, liberal in sentiment, liberal in every relation of life. This is that genuine liberality so rarely met with in the world. In the practice of his profession it was carried to a blameable extent. To refuse to wring, in sickness, their hard earnings from the indigent—to spare the blush, whilst we also spare the purse of decent poverty—to consult with considerate kindness the means of those whose lot is to sustain that hardest of struggles, the maintenance of a certain position in society with very inadequate resources—to lend the helping hand to the infirm of our own body—these are the privileges of the medical profession, which constitute its proudest boast, and will, we trust, be its unvarying practice. All this Dr. Johnson did, and more. Indiscriminate in generosity, he refused fees, where fees were no object to their owners, and where the money so rejected would be spent on frivolity or vice. For such a course no valid reason can be urged, and it is open to positive censure. It leads to imposition on good nature, (that is of little consequence)—but it leads also to the depreciation of medical remuneration generally. If a physician or a surgeon of distinction pitches his fees habitually low, he either undersells his brethren, or he puts them in the position of appearing avaricious. Now if there be a fixed professional fee, and they demand no more, it is unfair to them, and to the whole profession, to make that fee seem an excessive one. We do not object to discrimination, nor to liberality, however great, governed consistently by circumstances. It is to liberality without discrimination, and without any fixed or certain principle to regulate it, that we refer, and we think it is open to criticism. If blame, however, attach, in this instance, to Dr. Johnson, it will sit lightly on his memory, it touches the head and not the heart, and, living or dead, we cannot but admire the generous motive, however we may think that generosity extreme.

If his early life had not taught him avarice, it taught him prudence and economy. His tastes were simple and unostentatious. The plainest of liveries, the gravest of carriages, the least expensive furniture, the least luxurious board, were his. Show he equally hated and laughed at. The traditions of the sea were remembered even in the metropolis, and he would often prepare his own scanty meal in his own private room. His character indeed was not unmixed with eccentricity, and if he entertained extreme opinions, it was against parade.

No man could have a more unfeigned horror of debt. If it was practicable to pay with ready money, he did so—if not, the terms of payment must be of the shortest possible extension. To owe was to him the most miserable thing in the world, and, at his death, his estate was found unencumbered to a degree that could hardly have been thought possible.

It has often been a matter of observation, that those who, in public, exhibit much vivacity, are apt, in private, to be dull or even melancholic. "As miserable as a comedian" is a proverb. The psychological explanation would not, perhaps, be difficult, but we need go no farther than the fact. Dr. Johnson was no exception to the rule. In conversation and society, lively, humorous, enjoying, as it seemed, the moment, it could hardly be supposed that this was an irksome strain upon his spirits, and that he sought, in quiet and in solitude, the repose more congenial to his disposition. This tendency had, no doubt, been generated by his literary occupations, and habit confirmed what necessity began. But in the exercise of his profession he was always the same, and as he obtained some degree of success in it, perhaps it may not be unprofitable to others to learn what procured popularity for him.

The practice of physic brings us into contact with the distresses and miseries of life. We do not, as the calling of some men compels them to, appear to aggravate or insult wretchedness, but our mission is to console, and to assist. A cheerful manner—a kindly sympathy—a patient attention to those trifles, which, unimportant and tedious though they be, constitute the sick man's world—an evident desire to relieve—and that confidence in our own resources, whose source is knowledge, and which is recognised at once by the anxious and the eager eye of suffering,—these seem to be the qualities that should meet in the practical physician, and these were the qualities that met in Dr. Johnson. His was not the exaggerated kindness which caricatures reality, and assumes the ready tear, the convulsive grasp, the whining tone. It was a cheerful and a manly sympathy, one of acts as well as words, and none ever doubted its sincerity.

It came from the heart, and it went to it. Probably no man, since Dr. Baillie, has been more loved by his patients, and, after his death, their grief was both widely and loudly uttered.

As a practical man, he was ready and sagacious in opinion, decided though cautious in action. The larger portion of his practice, dealt, from its consulting character, in chronic cases. In these he was remarkably successful. An objection was often made to his prescriptions, that they were complicated and unchemical. He laughed at the criticism, and retained the habit, observing that he found his prescriptions answer, and that was the main consideration. In this there was undoubtedly much truth, for we have ourselves witnessed the excellent results of some of these elaborate formulæ.

For fifteen or twenty years, Dr. Johnson had been subject to attacks of gout, which, though not of a violent description, contributed to annoy and debilitate him. For the last two years of his life, these attacks became less frequent, indeed they almost disappeared. That proved to him, as it generally does, an unpropitious omen. In the Winter of 1844, he sensibly exhibited those changes of countenance and manner, which give, in advanced life, so significant a warning. During the greater part of 1845, he was ailing. There was no illness, but he looked ill. The cheek grew more pallid, the features more contracted, the stoop became perceptible, the limbs attenuated, the appetite was more capricious, the power of enjoyment was impaired. A tour was pressed on him, but he could not, or he would not, consider himself equal to it. He had for many years slept at a house which he had built on the northern confines of the Regent's Park. In the latter part of the Summer and Autumn, he took a cottage at Norwood, to which he retired in the afternoon, returning to town in the morning. At first he found this of service, but discovering or supposing that the water disagreed with him, he left Norwood in September, and went back to the Regent's Park. He had long been partial to Brighton, and the railroad offered him facilities of which he determined to avail himself. He resolved to remain there for a couple of months, coming up to town three days in the week to see his patients. Unfortunately, Brighton was so full that he found it impossible to procure a house or apartments on West Cliff, and was compelled to repair to East Cliff, the lower portion of the town. Here the drains are frequently deranged, and it happened that they had been so at the house he selected, immediately before his visit to it. The residents and visitors had all been seized with diarrhœa, and this attacked himself and those attending him, almost on the instant that he entered it.

His intention to remain at Brighton three days in the week was not acted on. Devoured by ennui, he turned with satiety from the groups on the Parade, or the boats upon the beach, and after one attempt, he fled to the express train, and came to and returned from London daily. The excitement and exertion were too great. On the 4th October, returning by the train to Brighton, he was seized in it with a rigor. On arriving at his lodgings, he exclaimed to his wife, that unless he could procure a free perspiration this attack would be his death. That night he was delirious. The diarrhœa, which had never ceased, became aggravated, dysenteric—the low fever of the aged and exhausted was established on it—and worn out by purging, tormina, tenesmus, hiccup, he expired on the 10th, surrounded by his family, and sensible nearly to the last.

His death was the echo of his life. It was not embittered by regrets for the past, nor terrors for the future. He looked back with satisfaction and forward with tranquillity. Resigned to the will of God and cheerful, his care was for others, not for himself, for the personal wants of his children and attendants, not for his own sad necessities. When Dr. Hall and Dr. Yates, who were attending him, assured him he was better, he quoted Pope's well-known saying, that "he was dying of good symptoms;" and when they expressed their sympathy for his sufferings, he thanked them for their kindness, begged they would not trouble themselves about him, and added, with a melancholy smile, that "his old shattered frame was not worth repairing." Nor did his strong sense desert him, till close upon that last and saddest moment when all sense is fled. Suffering the extremity of anguish, those around him attempted the consolation that a merciful Creator would soon ease his pains. "I am not so childish," he observed, "as to imagine, that the laws of Nature will be changed for me. I have much still to endure." A little later, when a slight and but a slight deliriousness began to cloud his faculties, he was asked if he had any further wish, in the disposition of his property. He paused a minute, in reflection, and replied, "It is best not. I may be confused, and might direct what would not be advisable." It was known that he had a desire at his heart, but feeling that his mind was not as clear as it was wont to be, he refused it utterance—his powerful understanding and his sense of justice crowning his death bed.

At the time of his decease, he was within a few months of his 69th year. He had, on several occasions, predicted that he should not survive his 70th. He left behind him, to mourn the loss of one of the best of husbands and of fathers, a widow, four sons, and a daughter. Of his sons, the eldest, Mr. Henry James Johnson, is Senior Assistant Surgeon to St. George's Hospital, and Lecturer on Anatomy and Physiology. He retains the house in Suffolk Place, where his father resided for more than twenty years, and he practises there as a Consulting Surgeon. The second son, William John, is at the Bar, and a Fellow of Caius College, Cambridge. The third, Thomas Edward, is a Solicitor in Lincoln's Inn. And the youngest, Athol Wood, was recently house-surgeon at St. George's Hospital, and is preparing to take out his degree as a Physician.

If we look back on Dr. Johnson's career, we cannot but deem it a successful one. He started in life without money, friends or education. He was literally unknown. By his energy and talents he worked out for himself education, a profession, reputation, and a competency, if not a fortune. He gratified his tastes, while he raised his position. Fond of observation, he saw a large portion of the globe—and happy in describing what he saw, his writings were a source of emolument and honour. By taste, by circumstances, he was the child of literature, and literature was to him most bountiful—his name was noised abroad by his popular writings: his fame will live with his standard works. His earliest aspirations were to venture all in London—and, in London, at last, he ventured and won. Ambitious of a good rather than a great name, the general regret that waited on his loss, proves that that ambition has been gratified. If he dreaded anything it was the decrepitude and childishness of declining years—he was spared both; he practised his profession as successfully as ever to within a week of his death, and his faculties were sound to his last illness. He did not worship wealth, but he desired a sufficiency—he obtained it, bequeathing to his widow an easy independence, and to his children what will sustain, though not debauch, their industry. What he prized above all things was integrity and honesty, and he has left a name which is synonimous with both.

His ashes repose in the family grave at Kensal Green. Above them a monument of Sicilian marble, bears, or is like to bear, the following inscription:—

JACOBO JOHNSON,
Medico Præclaro
Literarum haud Sordido Cultori
Multis Amicis Flebili
Marito, Patrique Optimo
Uxor Filiique Dolentes
Hoc sacrant Monumentum.

